# Comparative Proteomics and Physiological Analyses Reveal Important Maize Filling-Kernel Drought-Responsive Genes and Metabolic Pathways

**DOI:** 10.3390/ijms20153743

**Published:** 2019-07-31

**Authors:** Xuan Wang, Tinashe Zenda, Songtao Liu, Guo Liu, Hongyu Jin, Liang Dai, Anyi Dong, Yatong Yang, Huijun Duan

**Affiliations:** 1 Department of Crop Genetics and Breeding, College of Agronomy, Hebei Agricultural University, Baoding 071001, China; 2 North China Key Laboratory for Crop Germplasm Resources of the Education Ministry, Hebei Agricultural University, Baoding 071001, China

**Keywords:** proteomes, iTRAQ, filling kernel, drought stress, heat shock proteins, *Zea mays* L.

## Abstract

Despite recent scientific headway in deciphering maize (*Zea mays* L.) drought stress responses, the overall picture of key proteins and genes, pathways, and protein–protein interactions regulating maize filling-kernel drought tolerance is still fragmented. Yet, maize filling-kernel drought stress remains devastating and its study is critical for tolerance breeding. Here, through a comprehensive comparative proteomics analysis of filling-kernel proteomes of two contrasting (drought-tolerant YE8112 and drought-sensitive MO17) inbred lines, we report diverse but key molecular actors mediating drought tolerance in maize. Using isobaric tags for relative quantification approach, a total of 5175 differentially abundant proteins (DAPs) were identified from four experimental comparisons. By way of Venn diagram analysis, four critical sets of drought-responsive proteins were mined out and further analyzed by bioinformatics techniques. The YE8112-exclusive DAPs chiefly participated in pathways related to “protein processing in the endoplasmic reticulum” and “tryptophan metabolism”, whereas MO17-exclusive DAPs were involved in “starch and sucrose metabolism” and “oxidative phosphorylation” pathways. Most notably, we report that YE8112 kernels were comparatively drought tolerant to MO17 kernels attributable to their redox post translational modifications and epigenetic regulation mechanisms, elevated expression of heat shock proteins, enriched energy metabolism and secondary metabolites biosynthesis, and up-regulated expression of seed storage proteins. Further, comparative physiological analysis and quantitative real time polymerase chain reaction results substantiated the proteomics findings. Our study presents an elaborate understanding of drought-responsive proteins and metabolic pathways mediating maize filling-kernel drought tolerance, and provides important candidate genes for subsequent functional validation.

## 1. Introduction

Field grown crops, similar to other sessile organisms, often endure numerous environmental instabilities throughout their life spans [1,2]. Such constant exposure hampers plant growth and development, consequently resulting in reduced crop yields [3]. Among the environmental stress factors (such as salinity, drought, freezing, heat, etc.), drought is the single factor that imposes the most severe limitations to agricultural production [4,5,6]. In the backdrop of a continually increasing global human population, heightened food demand, and continuing global climate change, drought is anticipated to increase in occurrence and intensity under future agricultural practice [7,8]. Therefore, understanding how crop plants respond to drought stress at the molecular level remains critical for guiding the genetic improvement in drought tolerance, so as to maintain sustainable higher productivity under such climate change conditions.

Maize (*Zea mays* L.) is the third most important cereal crop in the world after rice (*Orzya sativa* L.) and wheat (*Triticum aestivum* L.) [9]. Aslam et al. [10] have dubbed maize “a multidisciplinary crop” because of its multiple uses in the human food, animal feed, and fodder, as well as biofuel production [11,12]. However, maize production is under severe threat from drought stress. The crop is most susceptible to drought from the flowering stage to grain filling stage [13,14]. Kernel development in maize is well-characterized, with grain filling occurring from ~15 days post pollination (DPP) to ~45 DPP [15]. Maize kernels are particularly sensitive to the negative effects of drought stress during grain filling [16], a period also considered critical from an aflatoxin (*Aspergillus flavus* L.) resistance perspective [17]. Moisture stress at this stage could result in seed abortion and decreased productivity [18]. The numbers of kernels set and filled under drought stress account for most of the variation in maize grain yield under drought and directly affects the harvesting index. Additionally, kernels near the ear tip will often abort after several weeks of growth if they are drought-affected [7]. Therefore, minimizing moisture deficit and maintenance of an active supply of photo-assimilates at the grain filling stage are essential in reducing the effects of drought on kernel final weight.

To overcome environmental perturbations within their habitats, plants institute several adaptive strategies at different levels, ranging from physiological through metabolic to molecular [10,19]. Maintenance of water status and physiological activity of the plant cell is achieved through metabolic adjustment; plants synthesize and accumulate various protective molecules, such as antioxidant enzymes (peroxidase (POD), superoxide dismutase (SOD) etc.), polyamines, amino acids (predominantly proline and glycine), and some sugars [4,20]. To protect themselves against reactive oxygen species (ROS) and photo inhibition, plants activate osmoprotection via osmotic adjustment and antioxidant scavenging defense systems, aided by plant growth regulators [2,21]. At the molecular level, plants institute stress responsive proteins, transcription factors, and signalling pathways among other strategies to respond to drought stress [21]. These molecules confer drought tolerance through protection of cellular contents or via regulation of stress responsive genes [10].

Although there have been a lot of studies [22,23,24,25,26] on the physiological, biochemical, and molecular bases of maize dehydration tolerance, most of them attached importance to the seedling and vegetative stage responses, and mostly employed cDNA micro arrays and transcriptome analyses. Thus far, very few studies [3,18] have focused on the proteomic analysis of maize drought stress response at the grain filling stage, despite this being the most critical stage at which soil moisture deficit stress has a direct effect on the final grain yield in maize [7,16], and despite proteomic analysis approach being more expedient to transcriptomic approaches [27,28].

With recent advances in the technologies and approaches in abiotic stress response evaluation, large scale, high-throughput proteomics has become a very powerful tool for performing comprehensive analysis of crop proteins and identification of stress responsive proteins in comparative abiotic stress studies [29,30]. Proteomics describes the study and characterization of a complete set of proteins present in a cell, organ, or organism at a given time (known as a proteome) [31,32]. Generally, proteomic approaches are useful for proteome profiling, comparative expression analysis of two or more protein samples, and localization and identification of post translational modifications (PTMs). Proteomics involves the detection of protein diversity, abundance, isoforms, compartmentalization, and interaction with other proteins [33]. Additionally, it provides for both qualitative and quantitative measurements of proteomes in specific plant tissues at specific developmental and physiological stages [32,34]. Protein profiles reflect changes in the protein expression of a given tissue and its cellular compartments in response to endogenous or external perturbations [35]. Besides being complementary to genomics, proteomics provides information on the molecular mechanisms underlying plant growth and stress responses, and is a crucial link between transcriptomics and metabolomics [29]. Whereas the structural and expressional variation identified at genetic or transcriptional levels is not always translated into the predicted phenotype because of post-translational modifications [36], the proteome (unlike the static genome) is dynamic, and the evaluation of proteins caters for PTMs, thus providing more knowledge in understanding biological functions [33].

Gel-free methods involving digestion of intact proteins into peptides prior to separation have now become very popular as compared to gel-based methods (such as 2DE), which have a low rate of throughput. Application of gel-free protein separation techniques and the next generation of proteomic techniques, including quantitative proteomics approaches, such as isobaric tags for relative and absolute quantitation (iTRAQ) and isotope-coded affinity tags (ICAT), have become widely employed in descriptive and comparative plant abiotic stress adaptation proteomic studies [37,38]. Particularly, the iTRAQ-based method provides a gel-free shotgun quantitative analysis and uses isobaric reagents to label tryptic peptides and to monitor relative protein and peptide mass tolerance (PMT) abundance changes [39]. It also provides for multiplexing of up to eight samples in a single experiment, thereby allowing for the time-dependent analysis of plant stress responses or biological replicates in a single experiment [40]. The technique has become widely useful in plant abiotic stress response studies [36,41,42,43].

Previously [44], we employed an iTRAQ-based method to evaluate, through comparative analysis approach, the seedling-stage responses of two contrasting maize inbred lines (drought tolerant YE8112 and drought sensitive MO17) to drought stress. We realized that YE8112 was comparatively more tolerant to drought stress than MO17 owing to its enhanced activation of photosynthesis proteins involved in thermal dissipation of light energy, enhanced lipid metabolism, improved cellular detoxification capacity, improved chaperon activities, as well as reduced synthesis of redundant proteins to help save energy for enduring stress [44]. Additionally, our transcriptomic study [45] suggested improved stress sensing and signaling, enhanced carbohydrate synthesis and cell wall remodeling, enhanced amino acid biosynthesis, as well as YE8112’s ability to sustainably accumulate greater amounts of proline and POD activity under water-limited conditions to be critical for drought tolerance.

In order to clarify the molecular mechanisms underlying maize response to drought stress at the kernel filling stage, herein we employed an iTRAQ-based approach to examine the protein expression profiles in developing kernels, and to compare the drought stress responses of the same (YE8112 and MO17) inbred lines after moisture deficit exposure for 14 days. Moreover, comparative phenotypic and physiological drought-stress response analyses buttress the proteomic analysis results. Our findings bring to light new insights into the principal responsive proteins and metabolic pathways and processes underpinning maize drought tolerance at the kernel filling stage. Additionally, building on our previous findings [44,45], we examine the similarities or differences between maize seedling and filling-kernel responses to drought in the context of gene and protein expression regulation and at the physiological level. Further, drought responsive proteins and genes identified herein could be harnessed for molecular breeding and biotechnological applications aimed at developing drought resilient cultivars.

## 2. Results

### 2.1. Inbred Lines Contrasting Phenotypic and Physiological Responses to Drought Stress

**Figure 1:**
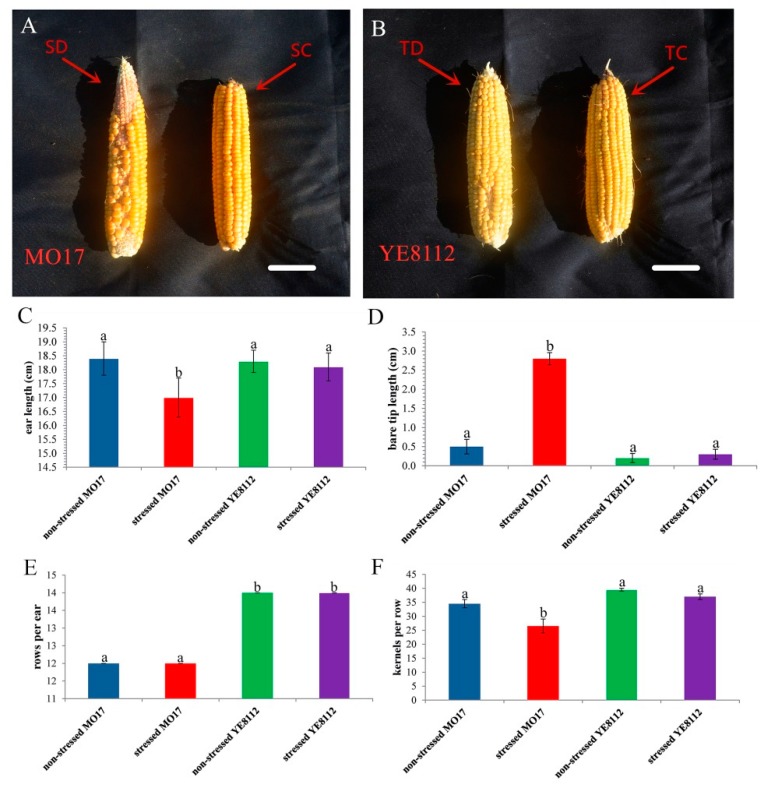
Phenotypic characterization of the two maize inbred lines (sensitive MO17, S; and tolerant YE8112, T) ears’ responses to drought stress. Observations and measurements were made at 26 days post pollination (DPP) under both water-sufficient (control, C) and water-deficit (drought, D) conditions. (**A**,**B**) Ear phenotypes; (**C**) ear length; (**D**) ear bare tip length; (**E**) kernel rows per ear; (**F**) kernel number per row. Data are presented as mean ± standard errors (*n* = 3). Different letters on error bars mean significant difference at *p* < 0.05. (**C**–**F**) Each replication is an average for the measurement of 10 ears. Scale bars = 4 cm for both Figure 1A,B.

At the 26 DPP stage, we recorded some phenotypic and morphological measurements of the sample ears from different treatment groups from both MO17 and YE8112 inbred lines. Phenotypically, under water-sufficient (control) condition, both inbred lines exhibited decent grain-filling condition and no prominent visual variances (sensitive line under water-sufficient conditions, SC in Figure 1A; tolerant line under water-sufficient condition, TC in Figure 1B).

However, under moisture-deficit conditions, significant differences in ear appearances were observed, with sensitive line MO17 showing uneven kernel arrangement and an obvious phenomenon of tip barrenness (sensitive line under drought treatment; SD in Figure 1A), whilst tolerant line YE8112 had even kernel arrangement and minimal barren tips (tolerant line under drought treatment; TD in Figure 1B). Drought stress resulted in significant (*p* < 0.05) decrease in ear length (EL) only in sensitive line MO17 (Figure 1C). Additionally, drought stress significantly increased ear tip barrenness in MO17, but not in YE8112 (Figure 1D). Meanwhile, relative to the control conditions, kernel rows per ear (KRPE) was not significantly influenced by drought treatment in both lines (Figure 1E). However, under drought conditions, kernels per row (KPR) was significantly (*p* < 0.05) decreased in sensitive line MO17, but not in tolerant line YE8112 (Figure 1F). Our results reveal vivid contrasting phenotypic responses of the two lines to drought stress at the kernel filling stage, with MO17 being comparably more susceptible than YE8112.

**Figure 2:**
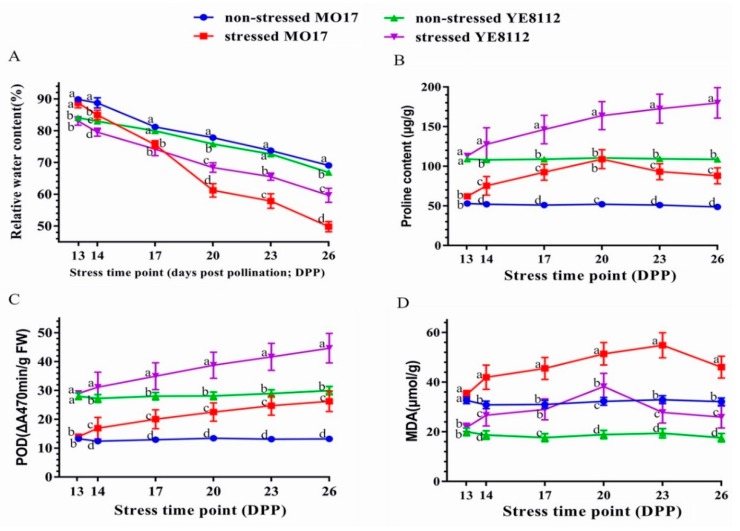
Physiological changes in the kernels of two contrasting maize inbred lines (sensitive MO17 and tolerant YE8112) in response to drought stress. Physiological changes were measured at different time points (13, 14, 17, 20, 23, and 26 days post pollination; DPP) under both water-sufficient (control) and water-deficit conditions. (**A**) Relative water content; (**B**) proline content; (**C**) peroxidase (POD) activity; (**D**) malonaldehyde (MDA) content. Data are presented as mean ± standard errors (*n* = 3). Different letters above line graphs show significant difference (*p* ≤ 0.05) among treatments at a given stress time point.

To understand the physiological responses of the maize plants to drought, we determined some physiological indices in the kernels. Compared to the control groups, the kernel relative water content (RWC) significantly (*p* < 0.05) declined in both inbred lines under drought stress, but the rate of decline in MO17 was much sharper than that of YE8112 (Figure 2A).

Our results showed that compared to the control conditions, proline content increased in both lines from 14 DPP to 20 DPP under drought conditions (Figure 2B). Proline increase was mantained up to 26 DPP in the tolerant line YE8112, wheras in the susceptible line MO17, it decreased from 20 DPP onwards (Figure 2B). Compared to control conditions, POD activity significantly increased in both inbred lines under drought stress conditions, starting from 14 DPP (Figure 2C). The rate of POD increase was statistically similar between the two lines (Supplementary Figure S1). Furthermore, as compared to the corresponding control, MDA content was significantly elevated by drought stress in both inbred lines. However, there was a sharp decline in MDA content in YE8112 from 20 DPP, and moderate decrease in MO17 starting from 23 DPP (Figure 2D).

### 2.2. Summary Inventory of Maize Filling-Kernel Proteins Identified by iTRAQ Analysis

The proteomes of MO17 and YE8112 kernels were collected under water-sufficient (control) and water-deficit conditions to make four libraries of three replicates each. The samples were then used for iTRAQ analysis. With the aid of Mascot software (version 2.2), 303, 288 spectra were matched with theoretical spectra from the Uniprot *Zea mays* L. database (132,339-12 January 2018). A total 50,566 peptides (comprising 27,288 unique peptides) corresponding to 6640 proteins (Supplementary Table S1) were identified. A broad coverage was observed on protein molecular weight (MW) distribution (Supplementary Figure S2A), with MW of the identified proteins ranging from 3 to 500 kDa. Among them, 162 (2.44%) weighed < 10 kDa, 5406 (81.42%) weighed 10–70 kDa, 634 (9.55%) weighed 70–100 kDa, and 438 (6.59%) weighed > 100 kDa (Supplementary Figure S2A). The number of peptides defining each protein is distributed in Supplementary Figure S2B, and over 77% of the total (6640) proteins matched with at least two peptides. In addition, the protein sequence coverage was generally below 30% (Supplementary Figure S2C). Further, the distribution of the peptide lengths defining each protein showed that most (90%) of the peptides ranged between 5 to 20 amino acids, with 7–9 and 9–11 amino acids as modal lengths (Supplementary Figure S2D). Proteins possessing at least two unique peptides were used in subsequent analysis of differentially abundant proteins (DAPs).

### 2.3. Analysis of Drought-Responsive Differentially Abundant Proteins (DAPs)

**Figure 3:**
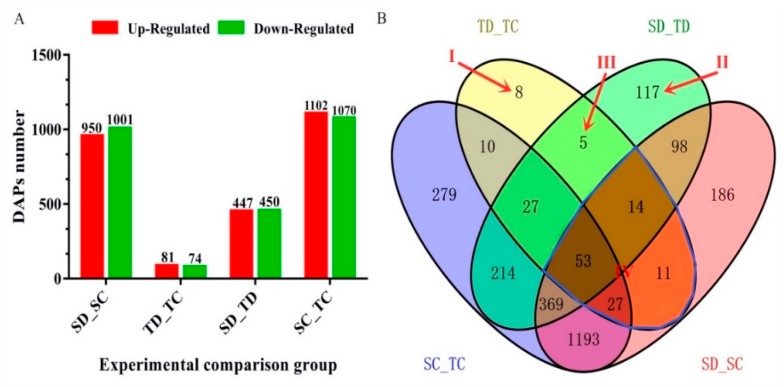
Analysis of differentially abundant proteins (DAPs) identified in four experimental comparisons. (**A**) Total number of DAPs identified in each experimental comparison group, by expression type. Up-regulated means DAPs with increased differential abundance. Down-regulated means DAPs with decreased differential abundance. (**B**) Venn diagram analysis of DAPs. Overlapping regions of the Venn diagrams indicate DAPs shared between or among corresponding groups. DAPs uniquely expressed in TC_TD (I), SD_TD (II), and TC_TD and SD_TD (III) are indicated with arrows. Area IV shows 105 overlapping DAPs within line.

We employed a comparative proteomic analysis approach to investigate the protein profile alterations in kernels of MO17 (drought-sensitive, S) and YE8112 (drought-tolerant, T) lines under water deficit conditions. A pairwise comparison of prior and post treatments (drought, D; control, C) was performed in MO17 (SC_SD) and YE8112 (TC_TD) individually. Additionally, we carried out a comparative analysis of the proteome between the sensitive and tolerant lines, under water-sufficient (control) (SC_TC) and drought (SD_TD) conditions, yielding four experimental comparisons (Figure 3A). A search for proteins with fold changes > 1.2 (up) or <0.83 (down) at *p* < 0.05 identified a total of 5175 DAPs among the four comparison groups. Prior to drought treatment, a total of 2172 DAPs were differentially expressed (SC_TC comparison), among which 1102 were up-regulated and 1070 down-regulated. Post drought exposure, 897 DAPs showed differential expression (SD_TD), with 447 being up-regulated and 450 down-regulated. Within the tolerant line YE8112, 155 proteins displayed differential abundance before and after drought treatment (TC_TD), with 81 DAPs being up-regulated and 74 down-regulated. Meanwhile, 1951 DAPs were observed in sensitive line MO17 before and after treatment (SC_SD), among which 950 were up-regulated and 1001 down-regulated (Figure 3A).

The Venn diagram (Figure 3B) displays a comparative analysis of the DAPs described above. The combinations of the four experimental comparisons reflect the impact of treatment or lines. With regards to drought tolerance, some combinations are more important than others. The sets of DAPs (marked Areas I–IV in Figure 3B) are the most critical and assumed more relevant to drought stress response. Among the 8 DAPs specific to YE8112 under drought stress conditions (Area I), half were up-regulated and another half down-regulated (Table 1). Of the 117 DAPs (Supplementary Table S2) unique to SD_TD (Area II; DAPs shared between sensitive and tolerant lines after drought treatment), 59 were up-regulated and 58 down-regulated (Supplementary Table S2). Meanwhile, the five DAPs unique to Area III are shown in Table 2. The 105 DAPs shared between TC_TD and SC_SD (Area IV), that is, the overlapping (common) drought-responsive DAPS within line, are listed in Supplementary Table S3. Additionally, the 186 DAPs uniquely expressed in sensitive line MO17 after drought treatment are listed in Supplementary Table S4.

**Figure 4:**
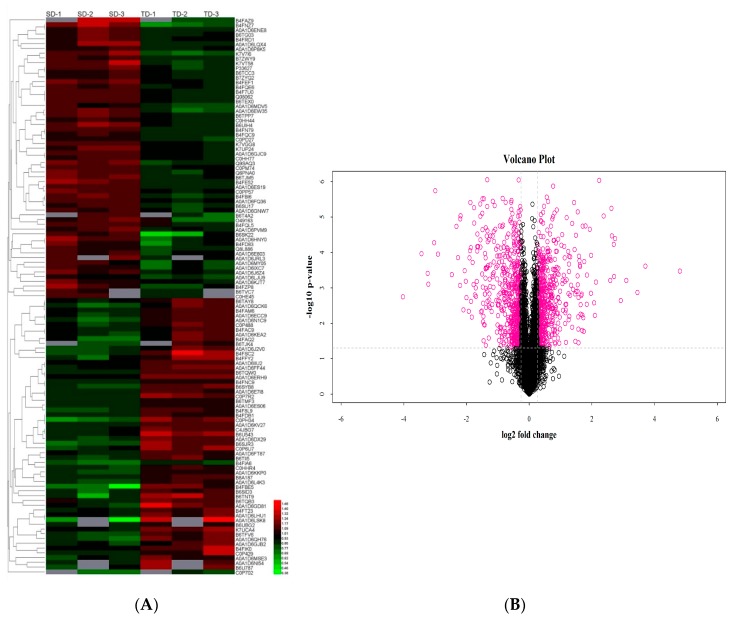
Clustering analysis of differentially abundant proteins (DAPs). (**A**) Heat map of DAPs overlapping in in SD_TD experimental comparison. Each row represents a significantly abundantly expressed protein. SD1-3 refers to the biological replicate number for MO17, whilst TD1-3 refers to the replicate number for YE8112. The DAPs were clustered based on the differentially expressed levels. The scale bar indicates the logarithmic value (log 2) expression of the DAPs, up-regulated (red) and down-regulated (green); (**B**) volcano plot showing the (log 2; −log 10 false discovery rate, FDR) expression of the DAPs in SD_TD comparison. Purple bubbles represent differentially expressed proteins and black bubbles represent proteins with non-differential expression.

We further conducted an overview hierarchical clustering analysis of the DAPs in the two inbred lines under drought stress conditions (proteins unique to SD_TD; Area II in Figure 3). Our results revealed that the DAP in different replicates of the same line exhibited similar expression patterns. However, comparisons of the same DAP in YE8112 and MO17 revealed differences in expression patterns (Figure 4A). Meanwhile, analysis of the differential expression changes of the DAPs showed that down-regulated and up-regulated DAPs were symmetrical (Figure 4B).

### 2.4. Functional Annotation and Classification of Drought-Responsive DAPs

We used Blast2GO web-based application (https://www.blast2go.com) to perform gene ontology (GO) functional annotation, that is, to assign level 2 GO terms to the identified DAPs. GO functional analysis revealed that several GO terms were shared between MO17 and YE8112, including metabolic process (GO:0008152), cellular process (GO:0009987), regulation of biological process (GO:0050789), and response to stimulus (GO:0050896), within the biological process (BP) category. Within the molecular function (MF) category, binding (GO:0005488), catalytic activity (GO:0003824), nutrient reservoir activity (GO:0045735), and transporter activity (GO:0005488) were the most prominent shared GO terms between the two lines. With regards to the cellular components (CC) compartmentalization, most of the DAPs in both inbred lines were located in the cell (GO:0005623), cell part (GO:0044464), membrane (GO:0016020), membrane part (GO:0044425), and organelle (GO:0043226) (Supplementary Figure S3).

**Figure 5:**
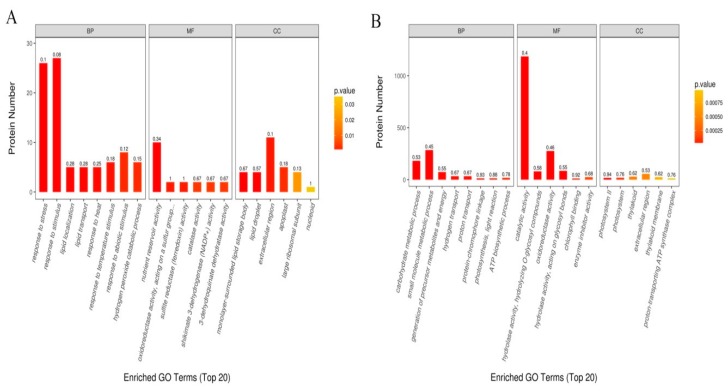
Gene ontology (GO) functional classification of differentially abundant proteins (DAPs). (**A**) Most significantly enriched GO terms (top 20) in tolerant line YE8112 under drought conditions; (**B**) most significantly enriched GO terms in sensitive line MO17 under drought conditions. The number above each bar graph shows the enrichment factor of each GO term (rich factor ≤ 1).

However, the analysis of the most significantly enriched top 20 GO terms in each line showed vivid differences in the GO terms between the two lines (Figure 5). In drought stressed YE8112, response to stress (GO:0006950), response to stimuli (GO:0050896), response to abiotic stimuli (GO:0009628), and hydrogen peroxide catabolic process (GO:0042744) were apparent under the biological process (BP) category. The GO terms nutrient reservoir activity (GO:0045735), oxidoreductase activity acting on a sulphur group (GO:0016667), and sulfite reductase (ferredoxin) activity (GO:0050311) were prominent in the molecular function (MF) category, whereas extracellular region (GO:0005576), monolyase-surrounded lipid storage body (GO:0012511), lipid droplet (GO:0005811), and apoplast (GO:0048046) were the apparent cellular component (CC) locations for the DAPs (Figure 5A). On the other hand, in drought stressed MO17, small molecule metabolic process (GO:0044281), cellular carbohydrate metabolic process (GO:0044262), and regulation of generation of precursor metabolites and energy (GO:0043467) were dominant terms in the BP category. In the MF category, catalytic activity (0003824), oxidoreductase activity (GO:0016491), and hydrolyase activity (GO:0016787) were most apparent. For the CC functions, photosystem II (GO:0009654) and thylakoid membrane (GO:0042651) were the most significantly enriched terms (Figure 5B). These differences in the most significantly enriched GO terms may be the main reason for the two inbred lines’ divergent drought tolerance, and hence arouse our keen interest for further discussion.

**Figure 6:**
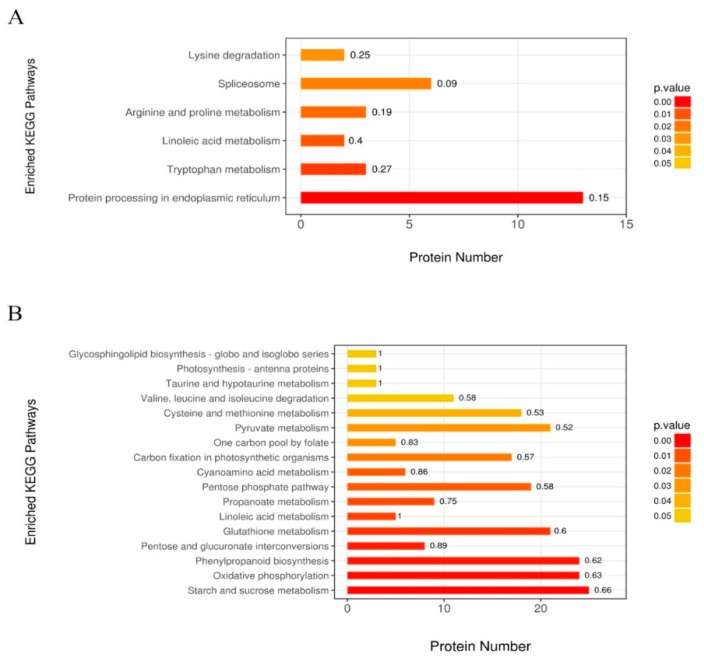
KEGG pathway enrichment analysis of the DAPs. (**A**) Most significantly enriched pathway in TD_TC; (**B**) most significantly enriched pathways in SD_SC, based on the hypergeometric test, *p* < 0.05. The color gradient represents the size of the *p* value; the color is from orange to red, and the nearer to red represents a smaller *p* value, and a higher significance level of enrichment of the corresponding KEGG pathway. The label above the bar graph shows the enrichment factor (rich factor ≤ 1).

To further analyze the functional fates of the identified DAPs, we mapped and assigned them to various metabolic pathways, based on the Kyoto Encyclopedia of Gene and Genomes (KEGG, available online: https://www.genome.jp/kegg/; accessed on 10 November 2018) database. We observed that the linoleic acid metabolism pathway responded to drought stress in both inbred lines (Figure 6), and that higher protein numbers were observed in MO17 enriched pathways than in YE8112 enriched pathways (Supplementary Figure S4). Using a hypergometric test, KEGG metabolic pathways with a *p*-value < 0.05 were labeled as significantly influenced by drought stress. Resultantly, protein processing in the endoplasmic reticulum and tryptophan metabolism pathways were the most significantly enriched in tolerant line YE8112 (Figure 6A). Contrastingly, starch and sucrose metabolism, oxidative phosphorylation, and phenylpropanoid biosynthesis pathways were the most significantly enriched in sensitive line MO17 (Figure 6B). For the full view of the top most significantly enriched pathways in YE8112 and MO17 lines, we refer you to Supplementary Figure S5. The diverse response exhibited by the two lines, in terms of the significantly enriched metabolic pathways, may be another clear contributing factor in their drought tolerance divergence.

### 2.5. Analysis of Protein-Protein Interactions

**Figure 7:**
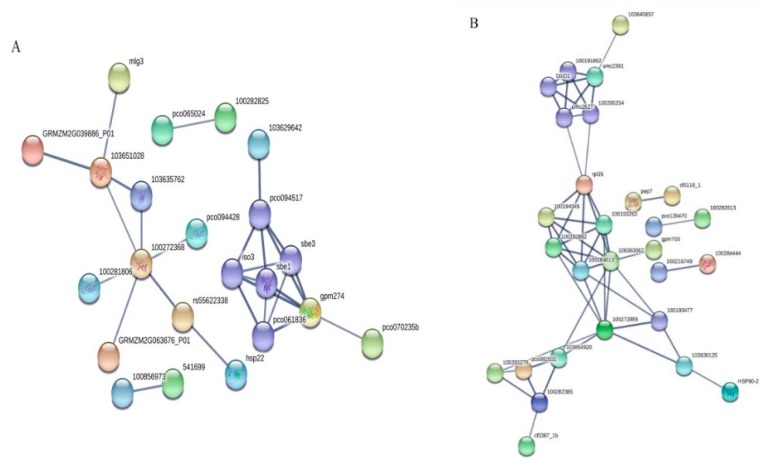
Protein–protein interaction analysis of the maize kernel drought-responsive differentially abundant proteins (DAPs). (**A**) DAPs differentially expressed in YE8112 after drought treatment (TC_TD). (**B**) MO17-specific DAPs. String database (version 10.5; http://www.string-db.org/) was used to construct the network. The nodes represent proteins, and the thickness of connectors between nodes represents the strength of the supporting data.

To understand how the maize kernel cells relay drought stress signals to influence certain cellular functions, the identified DAPs unique to YE8112 and MO17 inbred lines were further analyzed using the String database (Version 10.5, http://www.string-db.org/; accessed 10 March 2019). Selecting those DAPs with confidence scores higher than 0.7, two networks and two pairs of interacting proteins were observed in YE8112 (Figure 7A). The largest cluster comprises ten proteins, which are mostly heat shock proteins (Supplementary Table S5). The second group is made up of eight proteins; these participate in energy and carbohydrate (CHO) metabolism (Supplementary Table S5). Additionally, two protein pairs were identified to participate in drought stress response (Figure 7A), with the first pair being involved in ROS clearance and the second pair (of phosphogluconate dehydrogenases) involved in response to abscisic acid (Supplementary Table S5). Meanwhile, four distinct protein interaction networks were identified in MO17, comprising one sub-hub and three small clusters, as well as three protein pairs (Figure 7B).

### 2.6. Quantitative Real-Time RT-PCR (qRT-PCR) Analysis

**Figure 8:**
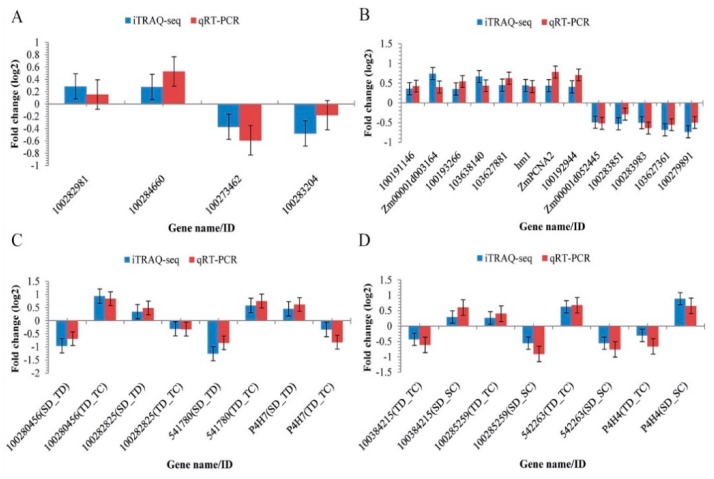
Quantitative real-time PCR (qRT-PCR) analysis results of the maize kernel drought-responsive genes encoding differentially abundant proteins (DAPs) from different experimental comparisons. (**A**) DAPs unique to TC_TD; (**B**) DAPs specific to SD_TD; (**C**) DAPs shared between SD_TD and TC_TD; (**D**) common or overlapping DAPs between TC_TD and SC_SD. All negative expression level values mean that the genes were down-regulated. *GAPDH* (accession No. X07156) was used as the house keeping gene. Error bars represent the SE (*n* = 3).

To confirm the accuracy of the iTRAQ results, the transcription of twenty-five representative genes (Supplementary Table S6) encoding proteins whose abundance was differentially expressed in response to drought stress was examined through a supporting quantitative real-time PCR (qRT-PCR) experiment. Resultantly, the transcription patterns and levels of all the twenty five sampled genes were consistent with our iTRAQ-based results (Figure 8; Supplementary Table S7). Thus, the qRT-PCR analysis results confirmed our proteomic analysis findings. Correlation coefficient analysis results (between qRT-PCR and iTRAQ data) are provided in Supplementary Figure S6.

## 3. Discussion

Drought or soil moisture deficit stress, particularly at the flowering and grain-filling stages, has devastating effects on the final yield in maize [7]. Therefore, understanding how maize plants respond to drought stress at the molecular level remains critical for guiding the genetic improvement in drought tolerance so as to maintain sustainable higher productivity under such climate change conditions. In order to gain comprehensive understanding of the molecular mechanisms, and identify putative proteome signatures associated with maize drought stress response at the kernel filling stage, in this manuscript, we have performed comparative proteomic analysis (between two inbred lines with contrasting drought sensitivity) using iTRAQ approach. Additionally, comparative phenotypic and physiological analyses of the two maize lines buttressed the proteomic analysis results. Our findings offer better insights into the molecular mechanisms underpinning maize drought tolerance at the kernel filling stage.

### 3.1. Vivid Contrasting Phenotypic and Physiological Responses of the Two Inbred Lines to Drought Stress

Significant differences were shown in ear appearances under moisture-deficit conditions, with sensitive line MO17 exhibiting more pronounced drought symptoms than tolerant line YE8112 (Figure 1A,B). Growth inhibition of ears and kernels was evident, with more significant decrease in EL and increased ear tip barrenness in MO17 than in YE8112 (Figure 1C,D). Further, the significant decrease in EL in MO17 was accompanied with corresponding reduction in KPR (Figure 1F). Drought stress results in cell growth arrest [10], slow ear growth [11], and fewer kernels [46]. The kernel-filling period is tightly linked to maize yield potential [17], with drought at this stage resulting in kernel abortion and yield reduction (emanating from diminished source strength and sink capacity) [10]. Retarded ear growth may sometimes be reflected by a failure to set kernels even when pollinated with sufficient fresh pollen [7]. Here, we suggest that drought stress decreased the remobilization of photosynthetic assimilates [4,47], causing a reduction in ear growth, kernel filling, and size, with more pronounced occurrence in the susceptible line MO17 [17]. Further, reduction in sucrose and starch synthesizing enzyme activities is also suggested to have resulted in corresponding kernel filling decrease [48]. Overall, our results revealed vivid contrasting phenotypic responses of the two lines to drought stress at the kernel filling stage, with MO17 being comparably more sensitive and less productive than YE8112.

Under environmental stress, reactive oxygen species (ROS), such as singlet oxygen (O_2_^−^) and hydrogen peroxide (H_2_O_2_), are greatly increased within cells. Excessive ROS will destroy biological macromolecules, such as protein DNA and lipids, resulting in strong oxidative effect on cells [49,50]. Our results showed that under drought stress conditions, there was a sustained increase in POD activities in both lines (Figure 2C). Peroxidases act to eliminate ROS and harmful substances within the cells. This helped the cells to cope with drought stress [51]. Proline can protect the integrity of cell membranes and reduce ROS damage to cells through stable osmotic regulation under abiotic stress [52]. With the increase of proline content in the cells, the water potential of the cells is reduced, thereby promoting absorption of water into the cells to cope with hydropenia [53]. Our results revealed that increase in proline content was sustainably maintained up to 26 DPP in YE8112 but decreased in MO17 from 20 DPP under drought conditions (Figure 2B). This could possibly point to why tolerant line YE8112 could endure drought stress better that sensitive line MO17. MDA content can reflect the degree of lipid peroxidation in the cells or tissue [54]. It is generally considered that the higher the MDA content in cells, the greater the damage to the membrane system. When the two inbred lines were exposed to drought stress, we observed that MDA content initially increased (until 20 DPP) in both lines, and then declined sharply in YE8112 (from 20 DPP), and gradually in MO17 (from 23 DPP) (Figure 2D). Increase in MDA content in both lines at the initial stages may suggest the damage of the cell membranes by ROS generation in the cells emanating from drought stress [41]. However, the sustained increase of proline content (combined with sustained increase in POD activity) beyond 20 DPP in tolerant line YE8112 might have aided better ROS quenching in YE8112 than in sensitive line MO17. Although sustained increase in POD activity was also witnessed in MO17, ROS could not be effectively quenched, probably as a result of lack of additional contribution by proline content, which started to decline 20 DPP. Consequently, drought stress caused a greater deal of damage to MO17 than to YE8112 kernel cells. In summary, we hypothesize that tolerant line YE8112’s better drought tolerance emanated from its enhanced ROS quenching ability, improved cell homeostasis regulation, better cell membrane integrity, and improved water retention capacity, as compared to sensitive line MO17 [44].

### 3.2. Differentially-Regulated Drought-Responsive Proteins in Tolerant Line YE8112

We performed comparative proteomic analysis using iTRAQ approach in order to identify putative proteome signatures in the tolerant maize inbred line YE8112. We observed that there was a pronounced change in the proteome of the stressed plants as compared to control conditions, as revealed by Venn diagram analysis (Figure 3B). We analyzed the iTRAQ data of YE8112 under drought conditions to screen out critical DAPs uniquely expressed in the tolerant line. Through bioinformatics analysis of these DAPs, proteins or genes and metabolic pathways responsive to drought stress were identified and clarified.

#### 3.2.1. Redox Post Translational Modifications (PTMs) and Epigenetic Regulation Mechanisms

Among the eight drought-responsive proteins that were expressed specifically in tolerant line YE8112 under drought stress conditions, half were up-regulated. Among these proteins were glyceraldehyde-3-phosphate dehydrogenase (GAPDH/G3PDH), cytosolic purine 5-nucleotidase, and histone deacetylase (Table 1). We hypothesize that these differentially up-regulated proteins are involved in drought stress tolerance in tolerant line YE8112. Plant GAPDH is a ubiquitous enzyme involved in glycolysis and has been proven a moonlighting protein, that is, to perform alternative non-metabolic functions, including transpiration control in Arabidopsis [55], phytopathogenic virus replication regulation [56], gene expression regulation, and oxidative stress sensing in plant cells [57]. Thus, these non-glycolytic roles of GADPH, via oxidative modifications of the catalytic cysteine, may be essential in drought stress response in maize. Cytosolic purine 5-nucleotidase in *Zea mays* L. cells is involved in metal ion binding, although its exact function remains unknown (www.uniprot.org/uniprot/A0A1D6L6C5). There is, therefore, need for its further characterization. Histone deacetylase (HDAC) is a key epigenetic enzyme for the binding of regulatory proteins to chromatin. Thus, histone deacetylation may represent a unique regulatory mechanism in the early steps of gene activation [58]. Taken collectively, these up-regulated proteins involved in redox PTMs and epigenetic regulation, via sequence-specific histone modification of genes, may be critical in drought tolerance in maize kernels.

#### 3.2.2. Response to Stress- and Response to Stimuli-Related Proteins under Drought Conditions

Our GO analysis of the DAPs expressed in YE8112 under drought (TC_TD) revealed that protein groups were enriched in “response to stress” and “response to stimuli” under the biological processes category (Figure 5). Among these proteins, eleven were up-regulated under drought stress (Supplementary Table S8, Sheet S1). These included several HSPs, chaperons, late embryogenesis abundant (LEA) protein group 3, defensin-like protein, and catalase, among others (Supplementary Table S8, Sheet S1). Under stress conditions, HSP70 plays a critical role in preventing aggregation and assisting in refolding of non-natural proteins [6,59]. The production of ROS and H_2_O_2_ under stress triggered the synthesis of HSP70, further enhanced the antioxidant enzyme activities, inhibited further accumulation of H_2_O_2_, and led to anti-stress and anti-apoptosis [60,61]. HSP90 is crucially involved in the management of protein folding [59,62], signal transduction networks, cell cycle control, protein degradation, and cellular protein transport [63]. Furthermore, as chaperones, small heat shock proteins (sHSPs) can protect proteins from aggregation under stress, thus promoting the cell’s anti-stress ability [64].

LEA proteins’ role in plant abiotic tolerance has been recognized due to their heat stability and hydrophilicity [6]; the group 3 LEA proteins accumulated in mature seeds and were regulated by abiotic stresses, such as dehydration, salinity, and low temperature [65]. LEA proteins particularly protect mitochondrial membranes against dehydration damage [66]. Zhang et al. [5] identified a group 3 LEA protein as a phosphorylated protein in two *Triticum aestivum* L. cultivars, with its phosphorylation level changing significantly under drought stress. Here, we suggest that the up-regulation of LEA group 3 proteins under drought stress helps protect YE8112 kernel cells and contributes immensely to maintaining cell homeostasis and normal energy metabolism. Taken collectively, we believe that when YE8112 is subjected to drought stress, it synthesizes more HSPs, enhances the chaperone functions of the protective proteins, maintains the cellular homeostasis, and improves the cell protection and tolerance to stress.

#### 3.2.3. Energy Metabolism and Secondary Metabolite Biosynthesis-Related Proteins under Drought

As we have already opined [45], carbohydrate or energy metabolism is critical for meeting the energy needs of a cell enduring drought stress. A number of enzymes related to CHO metabolism and secondary metabolite biosynthesis responded to drought stress in YE8112 inbred line kernels. The GAPDH, which plays a major role in the glycolysis, was up-regulated under drought (Table 1). Additionally, malate dehydrogenase (MDH), which is involved in CHO synthesis [67], the chalcone-flavonone isomerase family protein, involved in flavonoid biosynthesis, a part of secondary metabolite biosynthesis, and putative RUB1 conjugating enzyme and putative ubiquitin conjugation factor E4, involved in ubiquitin mediated proteolysis, were also up-regulated in response to drought stress (Supplementary Table S2). Further, abundance of other metabolism-related proteins, such as glycosyltransferase, galactose oxidase, inositol-1-monophosphatase, and proline iminopeptidase, also increased under drought conditions (Supplementary Table S2). These enzymes coordinate in carbohydrate energy metabolism and secondary metabolite biosynthesis and may be considered an essential drought response strategy in the tolerant line YE8112 kernels.

Besides, NADPH-dependent HC-toxin reductase (HCTR), which detoxifies the HC-toxin produced by the fungus *Cochliobolus carbonun*, and encoded by the maize *Hm1* gene [68] was also up-regulated in response to drought stress (Supplementary Table S2). This may suggest that the biotic-abiotic stress crosstalk mechanism was activated to holistically resist combined stress from drought and fungus, probably aflatoxin—which is most prevalent particularly at this physiological growth stage. Taken together, several proteins related to energy metabolism and defense response showed up-regulation 14 days after cessation of watering, suggesting that a global metabolic change occurred in maize kernels in response to drought stress. These proteins are suggested to play vital roles in maize filling-kernel drought stress response. Further research is needed to determine each of these proteins’ exact contribution in drought response.

#### 3.2.4. Seed Storage Proteins under Drought Stress

As revealed by our GO analysis results, nutrient reservoir activity (NRA) (GO:0045735) was the most significantly enriched level 2 GO term under the molecular function category (Figure 5A). Of the eight NRA-related DAPs that were significantly enriched in this GO term, six were up-regulated in response to drought stress. Among these were globulin-1 S allele, legumin 1, vicilin-like seed storage protein, Z1D alpha zein protein, and 50kD γ-zein protein (Supplementary Table S8, Sheet S1). Globulin and legumin are the main storage proteins in most angiosperms and gymnosperms [69]. Zeins are insoluble storage proteins with high proline content. The 50 kDa γ-zein plays an important role in the formation and stability of the protein bodies [70]. The up-regulation of 50kD gamma zein might have helped in reducing the dependence of maize kernels on water. Further, larger zein protein bodies ensure well filled kernel endosperm with a rigid matrix and lesser starch proportion [71]. Taken together, the up-regulated expression of seed storage proteins is hypothesized to increase the sink capacity of filling kernels and maintenance of intracellular homeostasis in drought-stressed YE8112.

#### 3.2.5. Most Significantly Enriched Metabolic Pathways of DAPs in Drought-Treated YE8112

The kernel proteome revealed changes in major metabolic pathways under drought conditions. Protein processing in endoplasmic reticulum (PPER), followed by ‘tryptophan metabolism’, were the most significantly enriched pathways in tolerant line YE8112 (Figure 6A). For the PPER pathway, eleven proteins were up-regulated, mostly HSPs (Supplementary Table S9, Sheet S1). These results resonate well with our GO analysis results where HSPs have been most significantly enriched in the prominent GO terms, suggesting their role as the major drought tolerance signature in YE8112 inbred line. Plants use the tryptophan pathway to provide precursors for the synthesis of secondary metabolites such as glucosinolates, and both indole- and anthranilate-derived alkaloids. Tryptophan metabolism, therefore, plays a direct role in regulating plant development and pathogen defense responses [72]. Here, we suggest that the tryptophan pathway may be involved in mediating biotic-abiotic cross talk in the kernels, thereby preventing cells from double trouble of drought stress and aflatoxin attack.

### 3.3. Differentially-Regulated Drought-Responsive Proteins in Sensitive Line MO17

For comparative analysis, in order to identify key drought stress-response differences between tolerant line YE8112 and sensitive line MO17, we also zeroed in on the most response strategies in MO17. Similar to our previous observation [44], tolerant inbred line YE8112 had fewer number of DAPs that were regulated in response to drought stress as compared to higher number in sensitive line MO17 (Figure 3). This may be probably because YE8112 perceived the drought treatment as moderate (as a result of its inherent better drought tolerance) and instituted limited proteomic response, whereas MO17, devoid of drought tolerance capabilities, perceived the same conditions as severe and hence deployed a heightened proteomic response.

#### 3.3.1. Plant Hormone Signal Transduction, Chaperone Activities and Protein Ubiquitination Processes under Drought Stress

Analysis of the sensitive line MO17 specific DAPs showed that indole-3-acetic acid (IAA), amido synthetase (GH3), three serine/threonine-protein kinase SRK2A, six chaperones, several ubiquitin carboxyl-terminal hydrolases (UCHs), and other ubiquitin-associated (UBA)/TS-N domain containing proteins, as well as proteasomes, were the most up-regulated in response to drought stress (Supplementary Table S4). GH3 catalyzes the conjugation of IAA to amino acids, an essential step in auxin homeostasis [73]. This may be vital for stress signal transduction. Serine/threonine-protein kinase SRK2A mediates plant hormone signal transduction (polar auxin transport) via the mitogen activated protein kinase (MAPK) signalling pathway [74]. Molecular chaperones play an essential mechanistic role of re-establishing normal protein conformation, and eventually cellular homeostasis in response to stress [75]. The UCHs, UBA-domain-containing proteins, and proteasomes have been implicated in proteosomal degradation, thereby regulating the proper protein turnover under stress conditions [44,76,77]. Taken together, these results suggest that a coordinated interaction between plant hormone signaling, chaperones, and ubiquitination processes forms a significant portion of maize drought responses.

#### 3.3.2. Decreased Mitochondrial Oxidative Phosphorylation Is Vital in Reducing Cellular ROS Generation in MO17

Analysis of the MO17 specific DAPs identified a total of 75 DAPs to be down-regulated in response to drought stress. Among these were NADH dehydrogenases, NADH ubiquinone oxidoreductases, and other enzymes involved in ATP synthesis (Supplementary Table S4). These enzymes play various roles in the oxidative phosphorylation process in the cells. NADH ubiquinone oxidoreductases can generate superoxide and H_2_O_2_ through multiple pathways, which are an important source of ROS [78]. In response, plant mitochondria prevent ROS generation within themselves via employing the alternative oxidase (AOX) pathway, in which complexes III and IV are bypassed and electrons are directly transferred to oxygen, resulting in thermal energy being generated instead of ATP [79]. A previous study has also shown that lack of NADH ubiquinone oxidoreductases will cause lower stomatal and hydraulic conductance so that the plants have better drought tolerance [80]. In the current study, the oxidative phosphorylation pathway of sensitive inbred line MO17 was greatly weakened as a result of drought stress, and was unable to supply sufficient energy. We believe that although the down-regulated expression of NADH ubiquinone oxidoreductases is conducive to reducing the accumulation of ROS, it was not enough to relieve the stress caused by drought stress on cells. Taken collectively, we herein infer that uncoupling of oxidative phosphorylation and electron transport is one of sensitive line MO17’s main drought stress response strategies enhancing the reduction of cellular ROS generation, although in this case, the phenomenon was not effective enough as a result of greater mitochondrial damage imposed by the 14-day drought treatment exposure.

#### 3.3.3. Most Significantly Enriched Metabolic Pathways of DAPs in Drought-Sensitive MO17

“Starch and sucrose metabolism” and “oxidative phosphorylation” were the most significantly enriched metabolic pathways in sensitive line MO17 (Figure 6B). Under drought conditions, the grain filling rate increased, but the duration shortened. However, the increased filling rate could not compensate for the shortened filling duration, resulting in decreased yield [81]. Nevertheless, plants under drought stress require a lot of energy to resist that stress and sustain their own metabolism. Studies have shown that speeding up carbon metabolism can provide more energy for stress resistance. However, enhancing energy production results in decreased carbohydrate synthesis, leading to a reduction in biomass [82].

Drought stress greatly limited the kernel filling process in MO17 (Figure 1A). At the initial stage of drought stress, the grout rate in MO17 was accelerated, but the loss of water in kernel cells led to the decomposition of starch and sucrose into soluble sugar to maintain the osmotic homeostasis and to supply a large amount of energy consumed by MO17, as well as enhancing signal transduction. We herein infer that the energy consumed by various pathways involved in MO17’s resistance to drought stress could not be compensated by enhanced assimilate remobilization, consequently contributing to the significant decline of MO17 yield under drought conditions. Nonetheless, our results may suggest that a coordinated activation of proteins or genes, enzymes, and pathways associated with starch metabolism and uncoupling of mitochondrial oxidative phosphorylation may be critical in effecting drought stress tolerance in sensitive maize inbred line MO17 filling kernels.

### 3.4. Similarities and Differences in Drought Stress Responses between Maize Seedlings and Filling Kernels

Comparative analysis of our previous [44,45] and current study findings revealed notable similarities and differences between maize seedling and filling-kernel drought stress responses in the context of gene or protein expression regulation and at the physiological level. “Photosynthesis antenna proteins” [44] and “nitrogen metabolism”, as well as secondary metabolites biosynthesis-related pathways (phenylalanine metabolism and phenylpropanoid biosynthesis) [45], were the most significantly enriched pathways in drought-stressed seedlings, whilst PPER and “tryptophan metabolism” were most significantly enriched in drought-stressed kernels. Meanwhile, according to the current study, filling kernel drought tolerance can be attributed to proteins involved in redox PTMs and epigenetic regulation mechanisms, elevated expression of HSPs, enriched CHO metabolism and secondary metabolites biosynthesis, up-regulated expression of seed storage proteins, and activated biotic–abiotic crosstalk mechanisms.

From the above findings, we deduced the following: (a) YE8112’s sustained maintenance of proline content and POD increase alongside decreased MDA content under drought stress conditions is essential for drought tolerance in both seedling leaves and filling kernels; (b) activated expression of HSPs, secondary metabolites biosynthesis, and CHO metabolism are universal in both seedlings and kernels, with differences only being noticed in the exact individual specific proteins or genes involved; (c) biotic–abiotic cross tolerance mechanisms were activated at both maize seedling and filling kernel stages, with non-specific lipid transfer proteins involved in plant bacteria and fungus attack response and long distance stress signalling being apparent in seedlings, whereas HC-toxin reductase—probably as a response to fungus *Aspergillus flavus* L. attack—was prominent in kernels; (d) enhanced thermal dissipation of light energy in photosynthesis machinery is critical for seedling leaf cells’ survival, whilst enhanced expression of seed storage proteins is essential in helping kernels endure drought stress; (e) even for a particular maize inbred line, a greater number of drought responsive proteins was observed in kernels than in the seedlings, highlighting the increased drought sensitivity of the kernel filling stage as compared to the seedling stage. Our combined results presented here will guide us in the selection of critical specific individual genes or proteins for targeted cloning and downstream analyses.

## 4. Materials and Methods

### 4.1. Plant Materials and Growth Conditions

Our laboratory (North China Key Laboratory for Crop Germplasm Resources of Education Ministry, Hebei Agricultural University, Baoding, China) provided the two drought tolerance divergent maize inbred lines used in this study. Previously, MO17 had shown high drought stress susceptibility, whilst YE8112 had shown high drought stress tolerance. For the detailed criterion on our selection of these inbred lines, we refer you to our recent study [44]. The field experiment was conducted at the Qing Yuan Experiment Station of Hebei Agricultural University, Baoding, China (115.560279′ E, 38.795093′ N, 118 m), under a fully automated rain-proof shelter. The experiment was set up in a randomized complete block design, with the control and drought stress treatment groups replicated three times. Each experimental unit or plot measured 5 m × 5 m, with plants spaced at 0.6 m × 0.3 m, giving 128 plants per plot. One week prior to anthesis, strict bagging was instituted in order to manage selfing of the plants during the anthesis stage. Both treatments received normal irrigation until the anthesis stage (at 12 DPP). At the grain filling stage (from 13 DPP to 26 DPP), drought stress treatment was then affected onto the treatment groups by withholding irrigation for 14 days, whereas the control group continued to receive adequate watering. Grain samples for physiological indices of assays were collected (from both control and drought treatment groups) at 14 DPP, and after every three days thereafter. Samples for proteomic analysis were collected at 26 DPP, from the middle part of each selected maize cob on the plant. At every sample collection, we ensured that the collected samples were immediately frozen in liquid nitrogen before storage at extremely low temperature (−80 °C) while awaiting subsequent use.

### 4.2. Phenotypic and Physiological Characterizations

The phenotypes and physiological characteristics of the grains of the two inbred lines at the filling stage were determined under both moisture-abundant and moisture deficit stress conditions. Ten ears were randomly selected from each group for the measurement of KRPE and KPR and the mean values were calculated. Further, we used visual observation to assess some phenotypic characteristics of the harvested cobs. The relative RWC of the kernels was estimated using the method developed by Lee [83]. The guaiacol protocol of Han et al. [84] was used to determine the activity of peroxidase (POD) in the kernels. Meanwhile, the content of free proline in the samples was determined using the ninhydrin chromogenic method developed by Bates et al. [85]. Lipid peroxidation was determined as the amount of MDA produced by the thiobarbituric acid reaction, as per the method developed by Dhindsa et al. [86].

### 4.3. Protein Extraction

Total proteins were extracted from the non-stressed and stressed kernel tissues of two maize inbred lines with three biological replicates (each containing 500 mg maize kernels) using the cold acetone method, as fully described in our recent paper [44]. Total protein concentrations of the extracts were determined as per the manufacturer’s instructions, using Coomassie Bradford Protein Assay Kit (23200, Thermo Fisher Scientific, Shanghai, China), with bovine serum albumin (BSA) as standard. The absorbance was determined at 562 nm using an xMark microplate absorbance spectrophotometer (Bio-Rad Laboratories Inc., Hercules, CA, USA) and protein extract quality was examined with tricine-sodium dodecyl sulfate polyacrylamide gel electrophoresis (SDS-PAGE) [87]. The protein samples were then stored at −80 °C.

### 4.4. Protein Digestion and Isobaric Tags for Relative and Absolute Quantification (iTRAQ) Labeling

Before protein digestion, reduction of disulfide bonds and alkylation of free cysteine residues were performed as previously described [88]. Each sample was put into a new tube and adjusted to 100 μL using 8 M urea mixed with 11 μL 1 M DTT. The sample was then incubated at room temperature (37 °C) for 1 h followed by vortexing at 4 °C and 14,000× *g* for 10 min. For alkylation, 120 μL 55 mM iodoacetamide was added to the supernatant and then incubated in a dark room for 30 min. Then, washing of the supernatant was achieved by using 100 μL 100 mM TEAB and centrifugation at 14,000× *g* for 10 min at 4 °C, followed by discarding the eluate. The washing step was repeated thrice.

Total proteins (100 µg samples) were digested using trypsin (Promega, Madison, WI, USA) at a ratio of protein:trypsin of 30:1 at 37 °C overnight (16 h) [88]. Post digestion step, the peptides were dried in a centrifugal vacuum concentrator and reconstituted in 0.5M TEAB. Applied Protein Technology Co. Limited (Shanghai, China) conducted the protein iTRAQ labeling process using an iTRAQ Reagents 8-plex kit (AB Sciex, Foster City, CA, USA) as per the manufacturer’s instructions. As described in our recent paper [44], one unit of iTRAQ reagent (defined as the amount of reagent required to label 100 µg of protein) was thawed and reconstituted in 70 µL isopropanol. The control replicates were labeled with iTRAQ tag 115 for drought-sensitive MO17 and tag 117 for drought-tolerant YE8112. The drought treated replicates were labeled with tags 114 and 116 for MO17 and YE8112 lines, respectively. Three technical replicates were performed.

### 4.5. Strong Cation Exchange (SCX) and LC-MS/MS Analysis

Sample fractionation was conducted before liquid chromatography-tandem mass spectrometry (LC-MS/MS) analysis using slightly modified procedures to those developed by Ross et al. [39]. Each SCX fraction was subjected to reverse phase nanoflow HPLC separation and quadruple time-of-flight (QSTAR XL) mass spectrometry analyses, as fully described in a previous report [43]. Peptides were subjected to nano electrospray ionization followed by tandem mass spectrometry (MS/MS). After fractionation, each 10 μL peptide was loaded onto an Eksigent nano LC System (AB SCIEX, CA, USA) with a trap C18 column (5 μm, 100 μm × 2 cm). Peptide elution was performed in an analytical C18 column (3 μm, 75 μm × 15 cm). The samples were loaded at 5 μL/min for 10 min before the 78 min LC gradient was run at 300 nL/min. The LC gradient started from 2 to 35% Buffer D (95% ACN, 0.1% FA) followed by the following schedule: 60% Buffer D at 5 min, 80% Buffer D at 2 min and maintained for 4 min, and finally return to 5% Buffer D in 60 s [88].

The mass spectrometry was analyzed by a Q-Exactive mass spectrometer (Thermo Fisher Scientific, Shanghai, China) after the sample had been analyzed by chromatography. The MS spectra with a mass range of 300–1800 *m*/*z* were acquired at a resolving power of 120 K, the primary mass spectrometry resolution of 70000 at 200 *m*/*z*, AGC (automatic gain control) target of 1 × 10^6^, maximum IT of 50 ms, and dynamic exclusion time (active exclusion) of 60.0 s The mass charge ratio of polypeptides and polypeptide fragments were set according to the following parameters: 20 fragments (MS2 scan) were collected after each scan (full scan), MS2 activation type was HCD, isolation window 2 *m*/*z*, two-grade mass spectrometry resolution of 17,500 at 200 *m*/*z*, the normalized collision energy of 30 eV, underfill of 0.1%. The electrospray voltage applied was 1.5 kV. Maximum ion injection times for the MS and MS/MS were 50 and 100 ms, respectively [44].

### 4.6. Protein Identification and Quantification

Mass spectrometry data from the LC-MS/MS raw files were obtained using Mascot software version 2.2 (Matrix Science, London, UK) and converted into MGF files using Proteome Discovery 1.4 (Thermo Fisher Scientific Inc., Waltham, MA, USA). The MGF files were analyzed against the Uniprot *Zea mays* L. database (132 339 sequences, 12 January 2018) using Mascot search engine. The following search parameters were set as follows: trypsin as the cleavage enzyme; two maximum missed cleavages allowed; fragment mass tolerance was set at ±0.1 Da; peptide mass tolerance was set at ± 20 ppm; monoisotopic as the mass values; iTRAQ 8 plex (Y) and oxidation (M) as variable modifications; and Carbamidomethyl (C), iTRAQ 8 plex (N-term) and iTRAQ 8 plex (K) selected as fixed modifications. Only peptides with *p* < 0.05 and false discovery rate (FDR) ≤ 1% were counted as being successfully identified [44].

As detailed in a previous report [88], protein relative quantification was dependent on the reporter ion ratios, from which relative abundance of peptides was estimated. Only proteins observed in all the samples were considered for quantification, with shared peptides being omitted. Peak intensities of the reporter ions were used for determining reporter ion ratios, with control-treated YE8112 sample serving as reference. Final protein quantification ratios were analyzed using the median average of those ratios; the median of unique peptide ratios represented the protein ratio. Fold changes were calculated as the average ratios of TD to TC and SD to SC, and the ratios for each protein in each experimental comparison were normalized to 1. Then, a paired *t-*test was used to compare the differences between the groups [43,88], and a protein was considered statistically significant at *p* < 0.05 and a fold change of >1.2 (up) or <0.83 (down) [3].

### 4.7. Functional Classification, Pathway Enrichment and Hierarchal Clustering Analyses of DAPs

The successfully identified DAPs were used as queries to search the Interpro (https://www.ebi.ac.uk/interpro/), Pfam (http://pfam.xfam.org/), Gene Ontology (GO, http://www.geneontology.org/), and the Kyoto Encyclopedia of Genes and Genomics (KEGG, http://www.genome.jp/kegg/) databases. Additionally, by searching the maize sequence database Gramene (http://ensemble.gramene.org/Zea_mays/), we obtained the corresponding gene sequences of the DAPs. The GO (protein) terms were assigned to each DAP based on BLASTX similarity (*E*-value < 1.0 × 10^5^) and known GO annotations using the Blast2GO tool (available online: https://www.blast2go.com; accessed on 15 January 2019). For the functional annotation and classification of the identified DAPs, GO analysis [89] was performed to categorize the DAPs into their BP, MF, and CC involvement in response to drought stress. Moreover, the DAPs were assigned to various metabolic pathways using the KEGG pathway analysis. Further, significant KEGG pathway enrichment analysis was performed using the hypergeometric test, with Q (Bonferroni-corrected *p*-value) < 0.05 set as the statistically significant threshold. Meanwhile, we used String web-based program (version 10.5) (http://www.string-db.org/) to construct a protein interaction network for the identified DAPs.

### 4.8. RNA Extraction, cDNA Synthesis, and qRT-PCR Analysis

Total RNA was extracted from non-stressed and stressed kernels of the two inbred lines (YE8112 and MO17) and prepared for quantitative real-time polymerase chain reaction (qRT-PCR) analysis using the Omini Plant RNA Kit (DNase I) (CWBIO, Beijing, China) as per the manufacturer’s protocol. To generate cDNA template, 1 µg of total RNA was reverse-transcribed in a total volume of 20 µL, using HiFiscript cDNA Synthesis Kit (CWBIO, Beijing, China) according to the manufacturer’s instructions. We randomly selected twenty-five DAPs and designed gene-specific primers (Supplementary Table S10) for qRT-PCR using Primer Premier 5 Designer software. Then, qRT-PCR was performed with a C1000 (CFX96 Real-Time System) Thermal Cycler (Bio-Rad), using 2 × Fast Super EvaGreen ^®^ qPCR Mastermix (US Everbright Inc., Suzhou, China). Each total 20 µL qRT-PCR reaction mixture comprised 1 µL of template cDNA, 1 µL of each primer (50 pmol), and 10 µL of 2 × Fast Super EvaGreen ^®^ qPCR Mastermix (US Everbright Inc., Suzhou, China). The amplification program was as follows: 95 °C for 2 min followed by 40 cycles of 95 °C for 10 s and 55 °C for 30 s [44]. A stable and constitutively expressed maize gene *GAPDH* (accession No. X07156), forward (GAPDH-F: 5′-ACTGTGGATGTCTCGGTTGTTG-3′), and reverse (GAPDH-R: 5′-CCTCGGAAGCAGCCTTAATAGC-3′) primers were used for housekeeping. The relative mRNA abundance was estimated using Livak and Schmittgen’s 2^−ΔΔ*C*t^ method [90]. Three biological replicates were performed for each sample.

### 4.9. Physiological Data Analysis

Student’s *t*-test was used to compare the data means of well-watered (control) and drought-treated plants of MO17 and YE8112 inbred lines. The statistical analyses of physiological data were perfomed with SPSS statistical package (Version 19.0; SPSS Institute Ltd., Armonk, NY, USA) using One-Way ANOVA, followed by Duncan’s multiple range test (DMRT) to evaluate the significant differences at *p* ≤ 0.05.

## 5. Conclusions

We have performed a comprehensive comparative iTRAQ proteomics analysis of maize filling-kernel proteomes of two inbred lines contrasting in drought tolerance exposed to 14 days drought treatment. In total, 5175 DAPs were identified from the four experimental comparisons. By way of Venn diagram analysis, four critical sets of drought-responsive proteins were screened and further analyzed by bioinformatics techniques. The DAPs uniquely expressed in YE8112 chiefly participated in pathways related to “protein processing in the endoplasmic reticulum” and “tryptophan metabolism”, whereas MO17 DAPs were involved in “starch and sucrose metabolism” and “oxidative phosphorylation” pathways. Most notably, our results revealed that YE8112 kernels were comparatively drought tolerant to MO17 kernels because of their enhanced redox PTMs and epigenetic regulation mechanisms, elevated expression of HSPs, enriched CHO metabolism, and up-regulated expression of seed storage proteins. Further, our comparative physiological analysis and qRT-PCR results substantiated the proteomics results. Our study presents an elaborate understanding of drought-responsive proteins and metabolic pathways mediating maize kernel drought tolerance, and provides important candidate genes for subsequent functional validation.

## Figures and Tables

**Table 1:** Drought-responsive maize kernel proteins identified specifically in tolerant line YE8112.

No	Accession ^1^	Gene Name/ID ^2^	Description ^3^	Covrg. ^4^	Pept. ^5^	Log_2_FC ^6^	*p* Value ^7^	Expr. ^8^	Pathway ^9^
1	A0A1D6N230	Zm00001d042191	Uncharacterized protein	11.16	6	1.234928	7.25 × 10^−4^	Up	
2	B6TDF8	100282981	Glyceraldehyde-3-phosphate dehydrogenase	61.07	19	1.219132	1.36 × 10^−4^	Up	Carbon fixation in photosynthetic organisms//Gluconeogenesis
3	A0A1D6L6C5	100284660	Cytosolic purine 5-nucleotidase	5.85	4	1.212466	6.72 × 10^−4^	Up	
4	B4FWF5	100273587	Histone deacetylase 6	18.38	2	1.211052	1.39 × 10^−3^	Up	
5	B6TPB9	100277111	Pentatricopeptide repeat-containing protein mitochondrial	16.47	6	0.824045	2.04 × 10^−2^	Down	
6	B4FVQ0	100273462	Pentatricopeptide repeat-containing protein mitochondrial	4.07	2	0.798673	3.42 × 10^−2^	Down	
7	B6U9Q8		mTERF family protein	5.52	2	0.773541	4.68 × 10^−2^	Down	
8	A0A1D6HT77	100283204	Galactose-1-phosphate uridyl transferase-like protein	7.34	2	0.717849	1.57 × 10^−3^	Down	Galactose metabolism//Amino sugar and nucleotide sugar metabolism

^1^ Accession = unique protein identifying number in the UniProt database; ^2^ gene name/ID = name or ID number of the corresponding gene of the identified differentially abundant protein DAP as searched against the maize sequence database Gramene (http://ensemble.gramene.org/Zea mays); ^3^ description = annotated biological functions based on Gene Ontology (GO) analysis; ^4^ Covrg. (%) = sequence coverage is calculated as the number of amino acids in the peptide fragments observed divided by the protein amino acid length; ^5^ Pept. = peptide fragments, refer to the number of matched peptide fragments generated by trypsin digestion; ^6^ Log2FC = fold change (log 2), is expressed as the ratio of intensities of up-regulated or down-regulated proteins between drought stress treatments and control (well-watered conditions). All the fold change values below 1 represents that the proteins were down-regulated; ^7^ *p* value = statistical significant level (using a paired *t*-test) < 0.05; ^8^ Expr. = gene expression level; Up = up-regulated; Down = down-regulated; ^9^ pathways = metabolic pathways in which the identified protein was found to be significantly enriched.

**Table 2:** Drought-responsive DAPs of the tolerant line that were also differentially expressed between tolerant and sensitive lines after drought treatment.

No	Accession	Gene Name/ID	Description	Covrg.	Pept.	YE8112 Fold Change		SD_TD Fold Change		Pathway
						Log2FC	*p* Value	Log2FC	*p* Value	
1	B6SHX8		Uncharacterized protein	32.43	3	1.618419	1.51 × 10^−3^	0.77178	2.65 × 10^−2^	
2	B6SGF3	100280456	Glyoxalase family protein superfamily	38.13	3	0.514965	3.06 × 10^−2^	1.912391	2.96 × 10^−2^	
3	B4FQG0	100282825	Hydrogen peroxide-induced 1	31.67	2	1.266202	4.56 × 10^−2^	0.807211	2.67 × 10^−2^	
4	B4FPJ4		ADP, ATP carrier protein	13.3	8	0.668951	3.03 × 10^−3^	1.308112	2.30 × 10^−3^	
5	B4FGT5	P4H7	Prolyl 4-hydroxylase 7	13.76	4	1.219904	4.69 × 10^−2^	0.825568	1.46 × 10^−2^	Arginine and proline metabolism

For detailed description of the columns, please refer to Table 1 caption above.

## Supplementary Materials

Supplementary materials can be found at https://www.mdpi.com/1422-0067/20/15/3743/s1.

## Abbreviations

CFI	Chalcone flavonone isomerase
DAP	Differentially abundant protein
DPP	Days post pollination
GAPDH/G3PDH	Glyceraldehyde-3-phosphate dehydrogenase
GO	Gene ontology
HCTR	HC-toxin reductase
HDAC	Histone deacetylase
HSPs; sHSPs	Heat shock proteins; small HSPs
iTRAQ	Isobaric tags for relative and absolute quantification
KEGG	Kyoto Encyclopedia of Genes and Genomes
LEA	Late embryogenesis abundant (proteins)
LC-MS/MS	Liquid chromatography-tandem mass spectrometry
MDA	Malondialdehyde
MDH	Malate dehydrogenate
NRA	Nutrient reservoir activity
POD	Peroxidases
PTMs	Post translational modifications
PPER	Protein processing in the endoplasmic reticulum
qRT-PCR	Quantitative real-time polymerase chain reaction
ROS	Reactive oxygen species
UCHs	Ubiquitin carboxyl-terminal hydrolases
