# Supplementary material for: Comparative Proteomics and Physiological Analyses Reveal Important Maize Filling-Kernel Drought-Responsive Genes and Metabolic Pathways
Source: Int J Mol Sci. 2019 Jul 31;20(15):3743. doi: 10.3390/ijms20153743 (PMC6696053; doi:10.3390/ijms20153743)
Supplement: Supplementary file 1 [file ijms-20-03743-s001.zip › Supplementary Figures.docx]

**Supplementary Figures – Total 6**

1a. Regression lines of peroxidase (POD) activity

1(b) ANOVA results of tests between the POD increase in MO17 and POD increase in YE8112


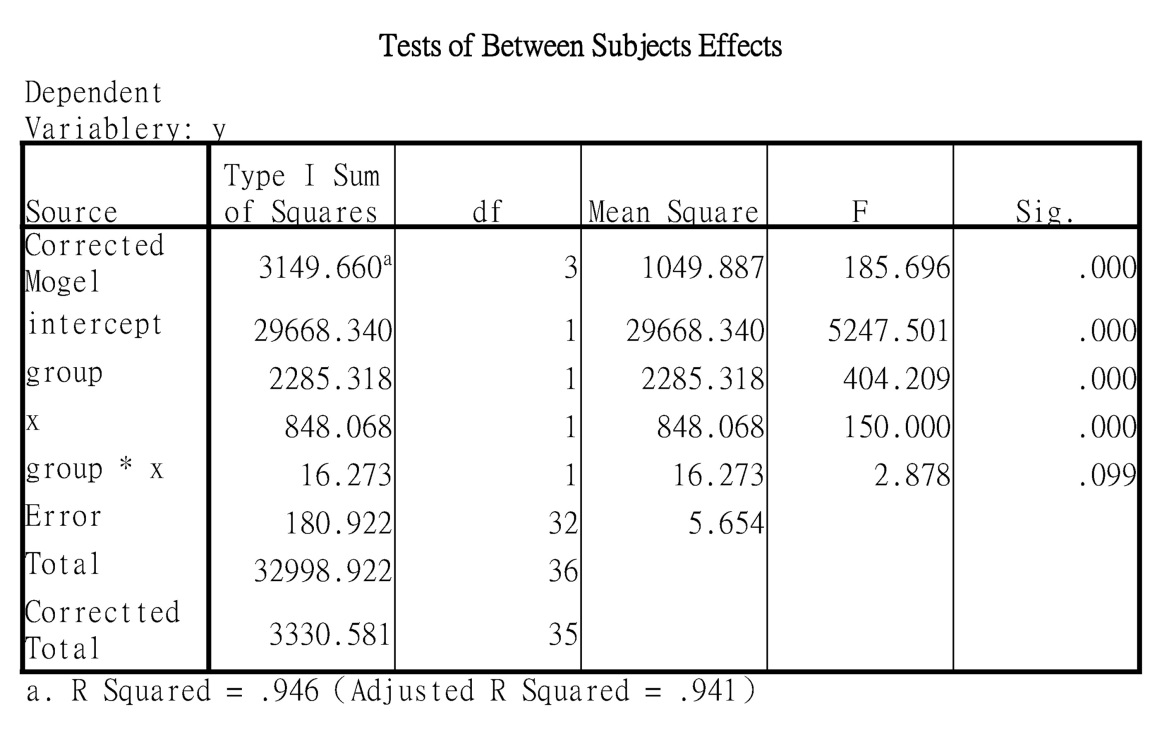


**Supplementary Figure 1. (A)** Regression lines/equations of peroxidase (POD) enzyme activity in contrasting maize inbred lines (drought sensitive line MO17 and tolerant line YE8112) under non-stressed (control) and drought stressed conditions. The X axis shows the drought stress treatment points (in days post pollination; DPP). The y axis shows the value of the measured parameter, that is µg/g. Each regression equation/line was calculated based on three replications, as highlighted by the legend. **(B)** The ANOVA table results of tests between the POD increase in MO17 and POD increase in YE8112. The results showed that the rate of POD increase in the two lines was not significantly different at the 0.05 level (Sig. 0.099 is above 0.05).


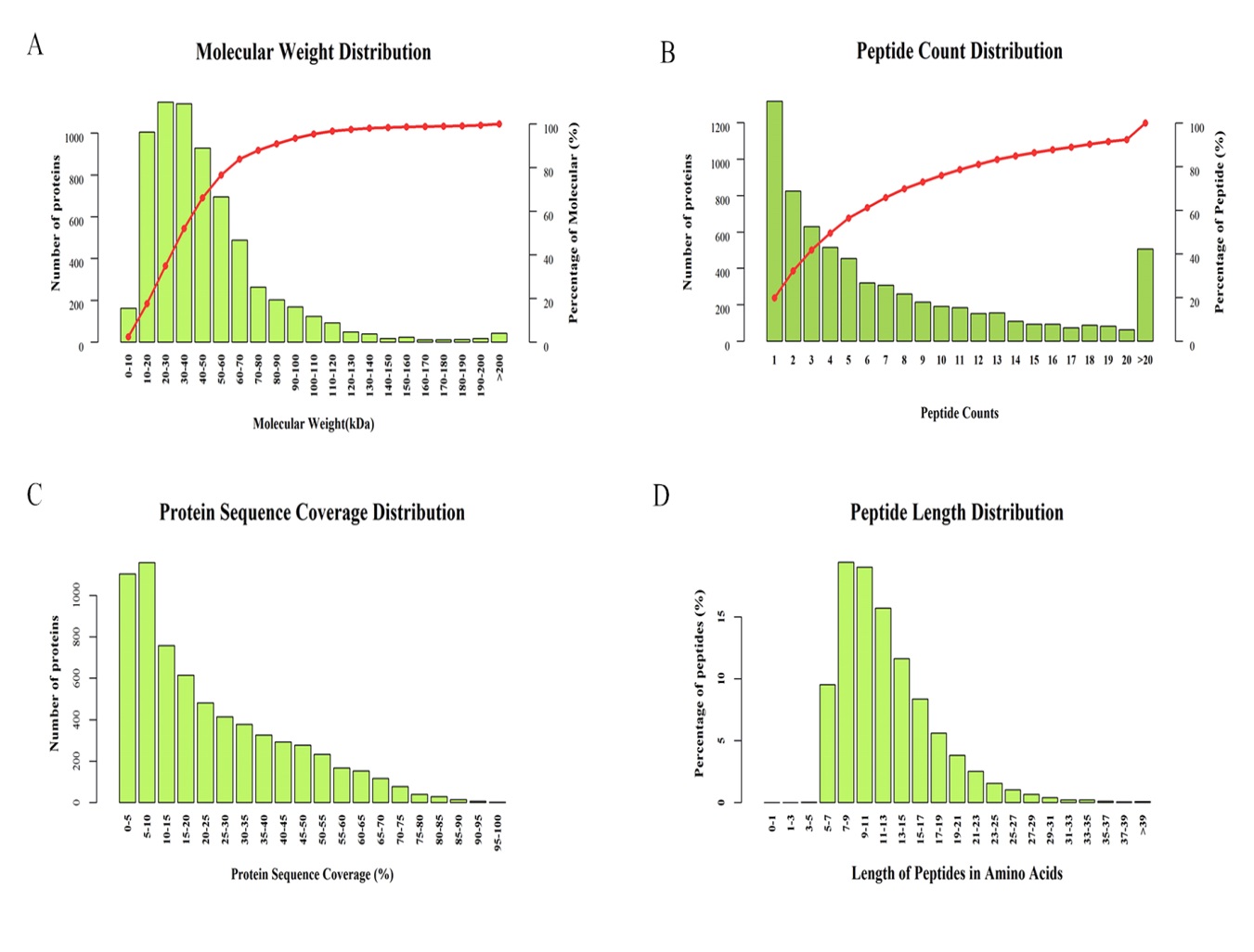


**Supplementary Figure 2.** Basic iTRAQ (isobaric tags for relative and absolute quantification) proteomics analysis output details. **(A)** Mass distribution of the identified proteins; **(B)** numbers of peptides that were matched to proteins; **(C)** sequence coverage range of the identified proteins; **(D)** length distribution of peptides defining each protein.

**
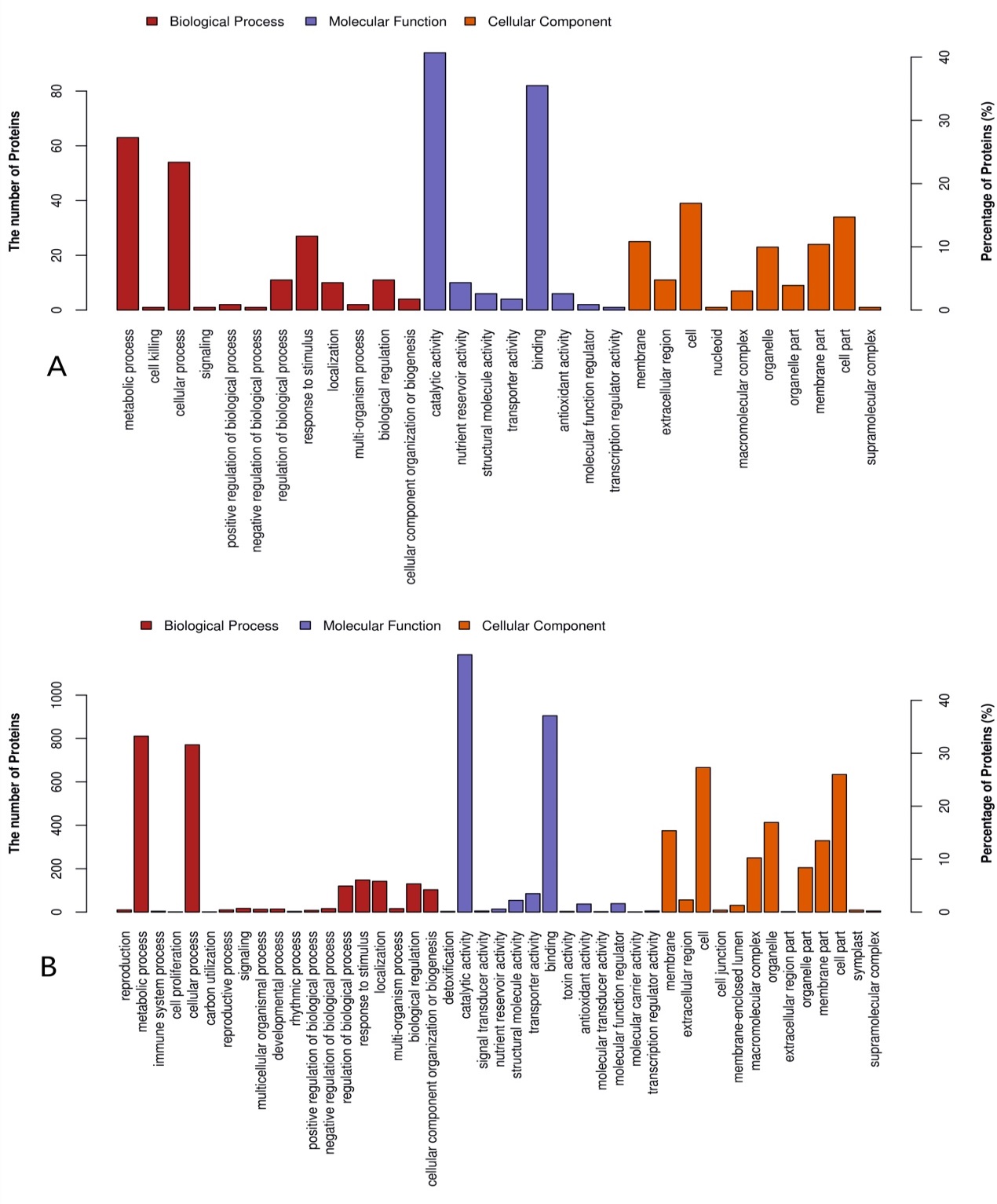
**

Supplementary Figure 3. Gene ontology (GO) functional classification of drought response proteins. (A) YE8112 specific DAPs; (B) MO17 DAPs, Y-axis represents the number of proteins in each function (and %); The X-axis shows the function of the protein, which is divided into three broad biological functional groups – biological process (BP), molecular function (MF) and cellular component (CC).


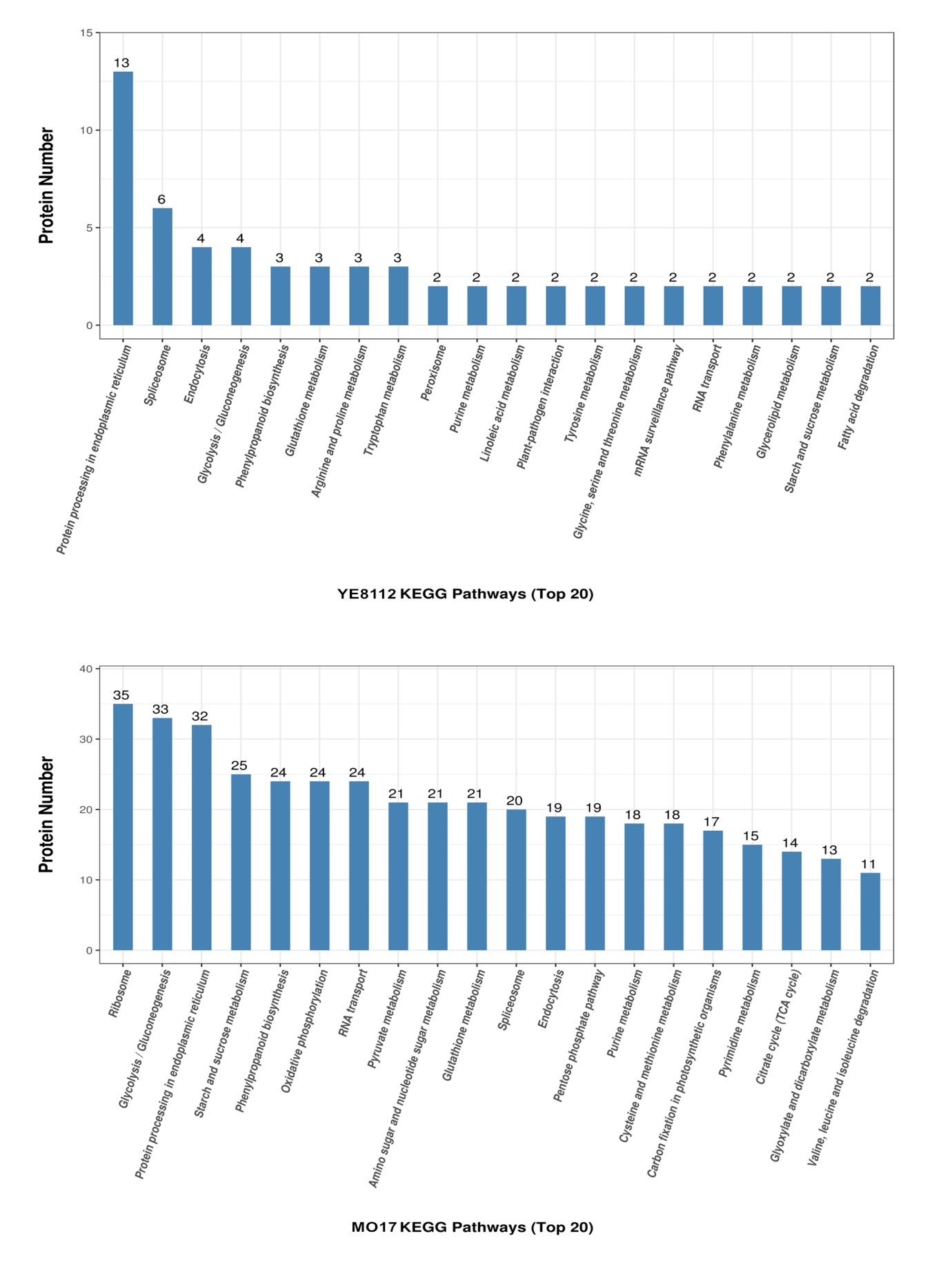


**Supplementary Figure 4**. Top 20 KEGG pathway enrichment analysis of the differentially abundant proteins (DAPs) in different experimental comparisons. (**A**) TC_TD comparison; (**B**) (**C**) SC_SD comparison. The whole number above the bar (blue) graph represents number of DEGs enriched in the corresponding pathway.

5a.

**
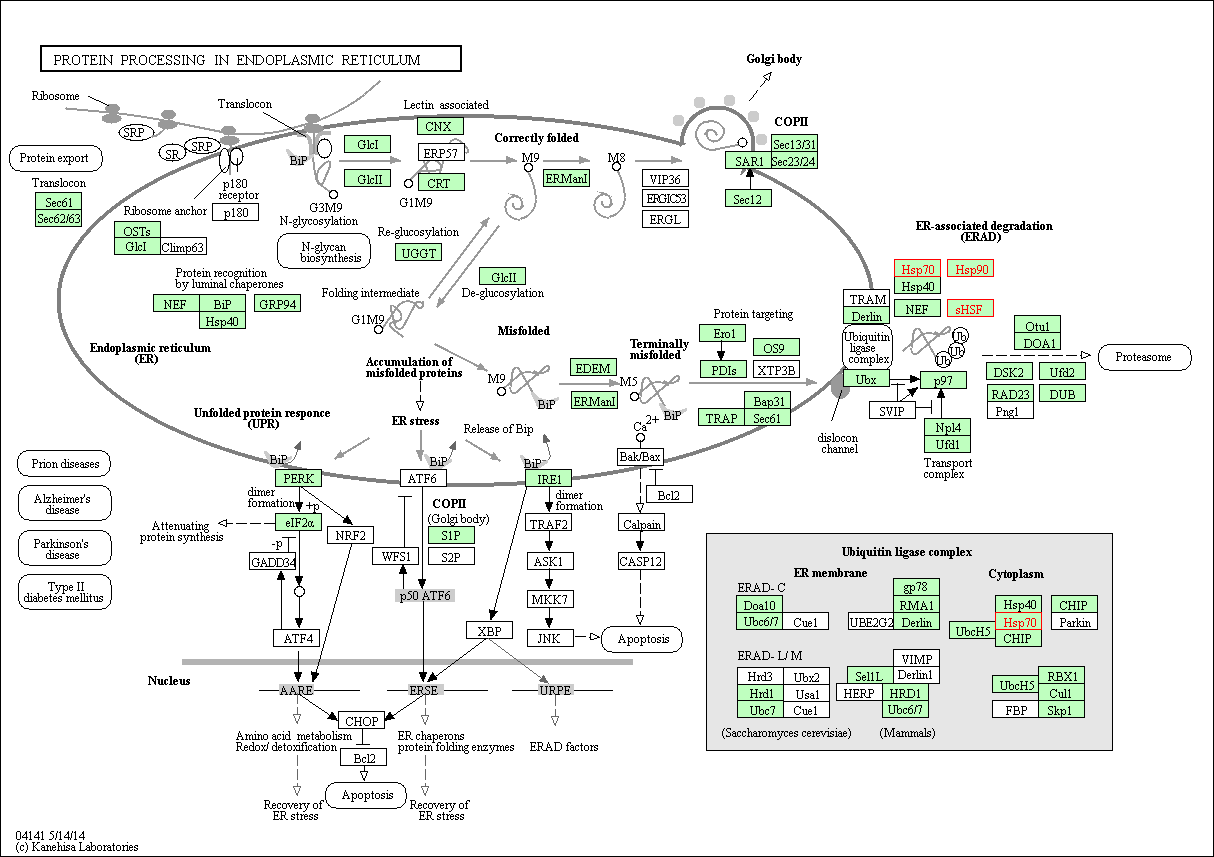
**

5b.


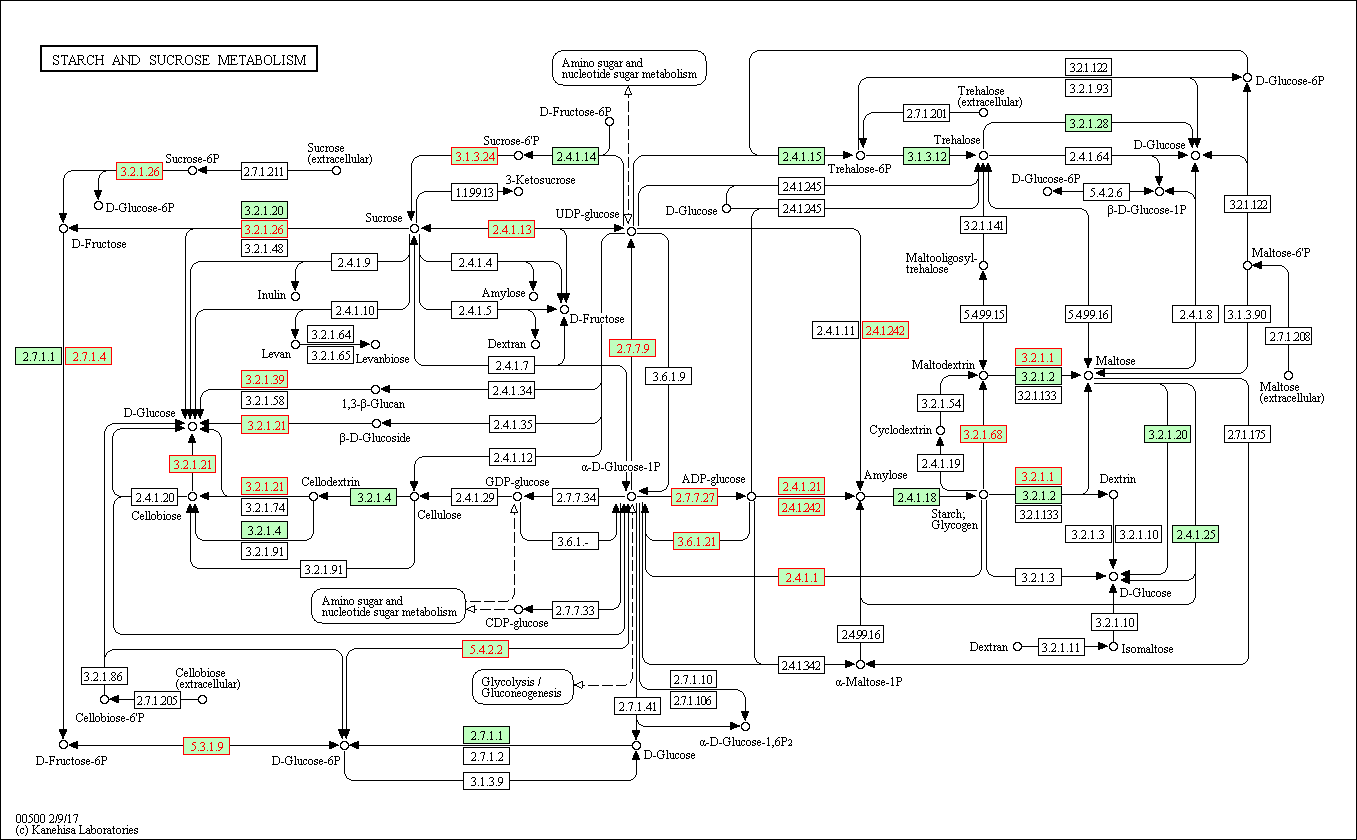


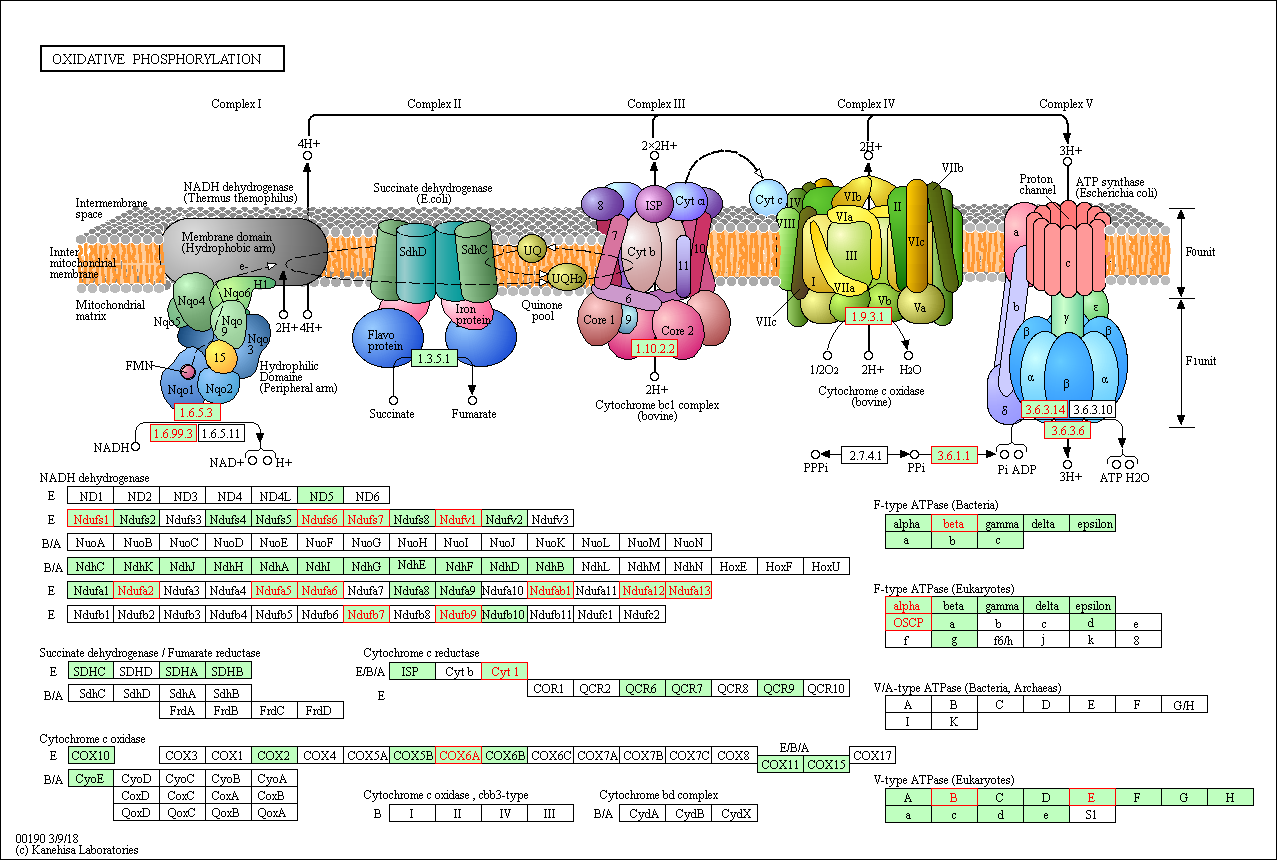


**Supplementary Figure 5.** Top most significantly enriched KEGG pathways in two inbred lines under drought stress conditions. The red boxes are proteins that were differentially expressed under drought conditions and the green boxes are substrates and metabolites. (A) ‘Protein processing in endoplasmic reticulum’ pathway was significantly enriched in tolerant line YE8112; (B) ‘Starch and sucrose metabolism’ and ‘Oxidative phosphorylation’ pathways were significantly enriched in sensitive line MO17.


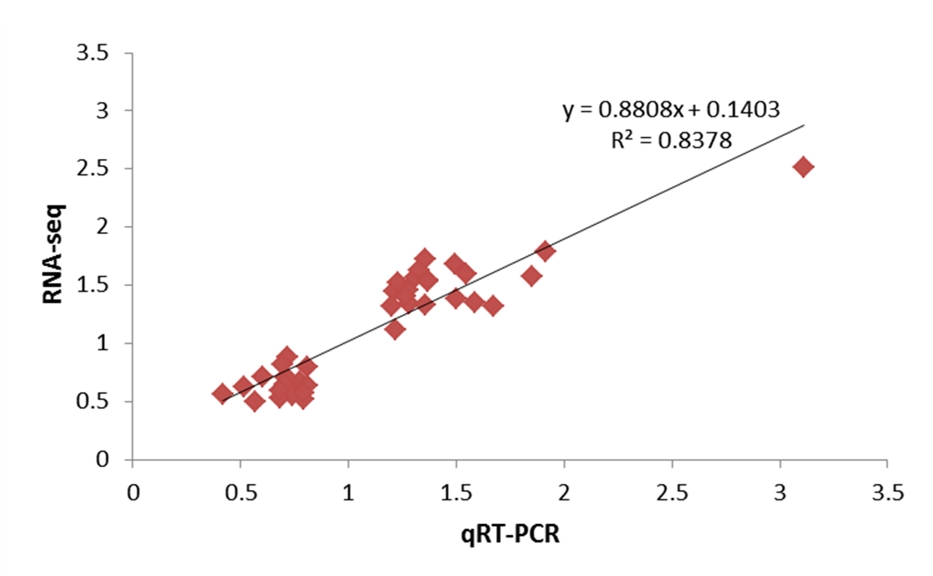


**Supplementary Figure 6.** Confirmation of iTRAQ-seq expression data through qRT-PCR analysis. Confirmation was performed using genes derived from four main groups of drought responsive DAPs identified in the study (See Tables 1-3 and Areas I-IV of Figure 4B in the manuscript). The plots demonstrate the expression ratio in Log scale with base of two. The X-axis indicates qRT-PCR Log scale; the Y-axis indicates RNA-seq Log scale.
