# Supplementary material for: Comparative Proteomics and Physiological Analyses Reveal Important Maize Filling-Kernel Drought-Responsive Genes and Metabolic Pathways
Source: Int J Mol Sci. 2019 Jul 31;20(15):3743. doi: 10.3390/ijms20153743 (PMC6696053; doi:10.3390/ijms20153743)
Supplement: Supplementary file 1 [file ijms-20-03743-s001.zip › Supplementary Tables/Supplementary Table 2.docx]

**Supplementary Table.** Drought-responsive maize kernel proteins identified specifically in SD_TD comparison

| No. | Accession^1^ | Gene Name/ID^2^ | Description^3^ | Covrg.^4^ | Pept.^5^ | Log^2^FC^6^ | p value^7^ | Expr.^8^ | Pathway ^9^ |
| --- | --- | --- | --- | --- | --- | --- | --- | --- | --- |
| 1 | B4FAZ9 | Zm00001d003164 | Ribonucleoside-diphosphate reductase small chain | 5.6 | 2 | 1.671356 | 0.003851208 | Up | Pyrimidine metabolism // Glutathione metabolism // Purine metabolism |
| 2 | B4FNZ7 |  | Peroxidase | 48.67 | 7 | 1.637676 | 0.00241145 | Up | Phenylpropanoid biosynthesis |
| 3 | K7V7I6 | 103638140 | Serine/threonine-protein phosphatase 2A 65 kDa regulatory subunit A beta isoform | 44.8 | 19 | 1.589153 | 0.001221147 | Up | mRNA surveillance pathway |
| 4 | B6SK22 |  | Chalcone-flavonone isomerase family protein | 20.89 | 4 | 1.568528 | 0.043589089 | Up | Flavonoid biosynthesis |
| 5 | A0A1D6EW35 | Zm00001d006422 | Peptide-N(4)-(N-acetyl-beta-glucosaminyl)asparagine amidase | 5.61 | 2 | 1.450424 | 0.003035219 | Up |  |
| 6 | K7VT58 | Zm00001d009567 | Fasciclin-like arabinogalactan protein 7 | 17.02 | 4 | 1.446257 | 0.011939846 | Up |  |
| 7 | B6T4A2 |  | Proteasome subunit beta type | 66.35 | 11 | 1.379773 | 0.029430373 | Up | Proteasome |
| 8 | A0A1D6HNY0 | 103627881 | Endoribonuclease | 9.92 | 5 | 1.365299 | 0.030476237 | Up |  |
| 9 | A0A1D6LQX4 | Zm00001d036756 | Uncharacterized protein | 50.51 | 3 | 1.364612 | 0.012491807 | Up |  |
| 10 | O49163 | hm1 | NADPH HC toxin reductase | 6.44 | 2 | 1.357294 | 0.046268545 | Up |  |
| 11 | Q9SAQ3 | ZmPCNA2 | Proliferating cell nuclear antigen | 57.79 | 14 | 1.353333 | 0.000188824 | Up | Nucleotide excision repair // Base excision repair // DNA replication // Mismatch repair |
| 12 | B4FE52 |  | Uncharacterized protein | 10.47 | 4 | 1.35174 | 0.001095694 | Up | Pyrimidine metabolism |
| 13 | B4FBI6 | 100192485 | Fumarylacetoacetase | 31.15 | 10 | 1.336996 | 0.002165237 | Up | Tyrosine metabolism |
| 14 | B4FZP8 |  | Uncharacterized protein | 16.46 | 3 | 1.334469 | 0.038255558 | Up | Monoterpenoid biosynthesis |
| 15 | B4FD83 | 100192944 | Alpha/beta-Hydrolases superfamily protein | 11.08 | 3 | 1.325715 | 0.047372971 | Up |  |
| 16 | Q6PNA0 |  | Putative RUB1 conjugating enzyme | 17.93 | 3 | 1.313385 | 0.001498612 | Up | Ubiquitin mediated proteolysis |
| 17 | B6UIH4 |  | ADP-ribosylation factor 3 | 42.57 | 5 | 1.302746 | 0.007366635 | Up |  |
| 18 | A0A1D6MY05 | 100191146 | Glycine-rich RNA-binding protein 3 mitochondrial | 34.12 | 4 | 1.28599 | 0.048436519 | Up |  |
| 19 | B4FEF1 | 100193266 | ATPase ASNA1 homolog | 33.06 | 10 | 1.277311 | 0.007248718 | Up |  |
| 20 | B7ZWY9 | 100279573 | Citrate synthase | 52.75 | 19 | 1.274647 | 0.000274474 | Up | Citrate cycle // Glyoxylate and dicarboxylate metabolism |
| 21 | B6TJM5 |  | 26S protease regulatory subunit 6A | 68.69 | 24 | 1.269335 | 0.002054366 | Up |  |
| 22 | A0A1D6IXC7 | Zm00001d024036 | C2H2-like zinc finger protein | 21.46 | 3 | 1.266202 | 0.040623203 | Up |  |
| 23 | B4F7U0 | 100191154 | Proteasome subunit alpha type | 58.13 | 14 | 1.26281 | 0.002506848 | Up | Proteasome |
| 24 | A0A1D6JRL3 | Zm00001d028050 | Retinoblastoma-binding protein-like | 3.16 | 2 | 1.260313 | 0.042307644 | Up |  |
| 25 | A0A1D6ENE8 | 100273017 | Guanosine nucleotide diphosphate dissociation inhibitor | 41.44 | 14 | 1.257962 | 0.00533938 | Up |  |
| 26 | B6SU17 | 100281287 | Phosphoglycerate mutase-like protein | 24.65 | 6 | 1.253306 | 0.001407596 | Up |  |
| 27 | Q08062 |  | Malate dehydrogenase, cytoplasmic | 76.51 | 20 | 1.251325 | 0.000687538 | Up | Carbon fixation in photosynthetic organisms // Cysteine and methionine metabolism // Glyoxylate and dicarboxylate metabolism// Pyruvate metabolism // Citrate cycle |
| 28 | P33627 | TUBA6 | Tubulin alpha-6 chain | 58 | 23 | 1.250024 | 0.01015871 | Up | Phagosome |
| 29 | C0HE45 | Zm00001d024938 | Peptidase S24/S26A/S26B/S26C family protein | 15.94 | 3 | 1.249969 | 0.015729832 | Up |  |
| 30 | A0A1D6J6Z4 | 100286133 | Nicotinate phosphoribosyltransferase-like protein | 3.54 | 2 | 1.24473 | 0.005351864 | Up |  |
| 31 | B4FN79 |  | Uncharacterized protein | 12 | 5 | 1.243918 | 0.00021149 | Up |  |
| 32 | B6TG03 | 100283230 | Ras-related protein RABA4a | 23.5 | 5 | 1.239268 | 0.003194824 | Up | Endocytosis |
| 33 | B6TVC7 | 100284586 | Ferredoxin | 27.88 | 3 | 1.23797 | 0.047740097 | Up |  |
| 34 | B6TPP7 |  | Threonine synthase | 34.67 | 15 | 1.236437 | 0.002580396 | Up |  |
| 35 | K7UP24 | Zm00001d048707 | Glycosyltransferase | 53.59 | 17 | 1.234922 | 0.009128778 | Up |  |
| 36 | C0PD27 | Zm00001d039079 | Isocitrate dehydrogenase [NADP] | 55.18 | 23 | 1.232344 | 2.72525E-05 | Up | Citrate cycle // Peroxisome // Glutathione metabolism |
| 37 | B4FQL5 | 100282671 | T-complex protein 1 subunit epsilon | 49.35 | 25 | 1.230696 | 0.024581291 | Up |  |
| 38 | A0A1D6P8K5 | 103639061 | Putative ubiquitin conjugation factor E4 | 8.6 | 8 | 1.226802 | 0.009861772 | Up |  |
| 39 | A0A1D6KJT7 | Zm00001d031599 | Proteasome subunit beta type | 35.62 | 6 | 1.226086 | 0.017589408 | Up |  |
| 40 | B6TCC3 |  | DNA-directed RNA polymerase II 36 kDa polypeptide A | 25.3 | 6 | 1.221233 | 0.002852051 | Up | Pyrimidine metabolism // Purine metabolism // RNA polymerase |
| 41 | A0A1D6ES19 | 103647533 | Heat shock protein 90-2 | 61.6 | 46 | 1.221075 | 0.000276467 | Up | Protein processing in endoplasmic reticulum // Plant-pathogen interaction |
| 42 | A0A1D6MDV5 | Zm00001d039146 | Carboxypeptidase | 14.13 | 6 | 1.218521 | 0.022544012 | Up |  |
| 43 | A0A1D6GJC9 | 103625953 | Phosphatidylinositol 4-kinase alpha 1 | 4.01 | 6 | 1.216253 | 0.004779356 | Up |  |
| 44 | C0PP57 |  | Uncharacterized protein | 26.43 | 12 | 1.214435 | 0.002646155 | Up |  |
| 45 | A0A1D6PVM9 | 100272774 | Regulator of Vps4 activity in the MVB pathway protein | 17.09 | 5 | 1.214309 | 0.028082578 | Up |  |
| 46 | B4FQE6 |  | Uncharacterized protein | 34.29 | 7 | 1.214279 | 0.001299761 | Up |  |
| 47 | C0HH44 | 100381568 | Pentatricopeptide repeat-containing protein | 7.79 | 6 | 1.213887 | 0.006429331 | Up |  |
| 48 | A0A1D6E803 | Zm00001d003259 | Galactose oxidase/kelch repeat superfamily protein | 5.43 | 2 | 1.213539 | 0.027140224 | Up |  |
| 49 | C0PM74 | 100384174 | Adenylate kinase 4 | 73.86 | 19 | 1.212295 | 9.01197E-05 | Up | Thiamine metabolism |
| 50 | B6TEX0 |  | Inositol-1-monophosphatase | 25.84 | 7 | 1.209535 | 0.000598498 | Up | Inositol phosphate metabolism // Phosphatidylinositol signaling system // Ascorbate and aldarate metabolism |
| 51 | C0HH77 |  | Uncharacterized protein | 17.99 | 4 | 1.208174 | 0.003218831 | Up |  |
| 52 | A0A1D6FQ36 | 100501867 | Guanosine nucleotide diphosphate dissociation inhibitor | 68.14 | 25 | 1.207467 | 0.002287345 | Up |  |
| 53 | K7VGG8 | Zm00001d010522 | ATP-dependent zinc metalloprotease FTSH 10 mitochondrial | 20 | 14 | 1.204039 | 0.001220634 | Up |  |
| 54 | B4FQC9 | 100272740 | Proline iminopeptidase | 64.6 | 17 | 1.203653 | 5.93084E-05 | Up | Arginine and proline metabolism |
| 55 | A0A1D6LJU9 | 103629390 | Plant/F18O14-17 protein | 6.16 | 4 | 1.203322 | 0.00369806 | Up |  |
| 56 | B4FRD1 | 100272882 | Adaptin ear-binding coat-associated protein 1 | 11.07 | 3 | 1.202934 | 0.020466063 | Up |  |
| 57 | A0A1D6GNW7 | Zm00001d013964 | ALG2-interacting protein X | 22.95 | 18 | 1.202627 | 0.008166607 | Up |  |
| 58 | B7ZYQ2 |  | Uncharacterized protein | 18.73 | 3 | 1.200388 | 0.004201136 | Up |  |
| 59 | Q8L886 |  | Nucellin-like aspartic protease (Fragment) | 34.73 | 11 | 1.20008 | 0.012930262 | Up |  |
| 60 | B6TAY8 | 100282744 | Delta(12)-fatty-acid desaturase | 14.8 | 4 | 0.832492 | 0.017203844 | Down | Biosynthesis of unsaturated fatty acids |
| 61 | B4FAC9 | Zm00001d014325 | Calcium-binding EF-hand family protein | 20.96 | 7 | 0.832446 | 0.037578906 | Down |  |
| 62 | B4FT23 | 100283140 | 14-3-3-like protein | 63.89 | 15 | 0.827561 | 0.023943663 | Down |  |
| 63 | A0A1D6IIU2 | 100282082 | Saccharopine dehydrogenase | 47.14 | 13 | 0.826533 | 4.62602E-05 | Down |  |
| 64 | B4FDB1 | 100192966 | NAD(P)-binding Rossmann-fold superfamily protein | 48.13 | 13 | 0.826238 | 0.000960073 | Down |  |
| 65 | C0P702 |  | Uncharacterized protein | 8.94 | 3 | 0.825969 | 0.03144713 | Down | Peroxisome // Glyoxylate and dicarboxylate metabolism |
| 66 | A0A1D6E7I8 | 100383448 | Putative E3 ubiquitin-protein ligase ARI8 | 22.24 | 9 | 0.823576 | 0.000365062 | Down |  |
| 67 | A0A1D6ES06 | Zm00001d005998 | Caffeoyl-CoA O-methyltransferase 1 | 45.56 | 10 | 0.822173 | 0.001250479 | Down |  |
| 68 | B6TMF3 |  | Wound/stress protein | 26.47 | 4 | 0.821963 | 0.000352102 | Down |  |
| 69 | B4FNC9 | 100281934 | Small ubiquitin-related modifier | 55.56 | 5 | 0.821764 | 0.000261649 | Down | RNA transport |
| 70 | B4FAM6 | Zm00001d034059 | 60S ribosomal protein L13a-1 | 45.15 | 10 | 0.821471 | 0.007896532 | Down |  |
| 71 | A0A1D6LHU1 | 100384506 | Pumilio homolog 3 | 3.21 | 3 | 0.82123 | 0.041532388 | Down |  |
| 72 | A0A1D6GJB2 | Zm00001d013441 | Coatomer subunit alpha-1 | 46.47 | 30 | 0.820364 | 0.047133975 | Down |  |
| 73 | A0A1D6FT87 | Zm00001d010727 | BAG family molecular chaperone regulator 8 chloroplastic | 4.57 | 2 | 0.819536 | 0.009153092 | Down |  |
| 74 | A0A1D6KKP0 | Zm00001d031688 | Putative 14-3-3 protein | 64.59 | 15 | 0.81724 | 0.00082948 | Down |  |
| 75 | B6TQB3 | 100284131 | Ran-binding protein 1 | 63.33 | 11 | 0.816963 | 0.013288628 | Down |  |
| 76 | A0A1D6ECC9 | 100284269 | Dihydrolipoyllysine-residue succinyltransferase component of 2-oxoglutarate dehydrogenase complex | 58.36 | 18 | 0.816644 | 0.016114126 | Down |  |
| 77 | B4FIA6 | 100194327 | Histone H2A | 34.81 | 4 | 0.815166 | 0.021339721 | Down |  |
| 78 | C4JBG7 | 100284902 | 3-isopropylmalate dehydratase large subunit | 49.9 | 20 | 0.813704 | 0.002492977 | Down |  |
| 79 | C0HHR4 | 100381643 | Actin-7 | 63.4 | 18 | 0.812995 | 0.006880468 | Down |  |
| 80 | C0P488 | 100382091 | Polyadenylate-binding protein RBP45C | 7.39 | 3 | 0.811061 | 0.036505416 | Down |  |
| 81 | B6UBG2 | 100282624 | Protein transport protein SEC13 homolog B | 32.13 | 6 | 0.810271 | 0.003631978 | Down | RNA transport // Protein processing in endoplasmic reticulum |
| 82 | B8A187 | Zm00001d025166 | Putative quinone-oxidoreductase homolog chloroplastic | 48.67 | 14 | 0.810013 | 0.001290995 | Down |  |
| 83 | A0A1D6KEA2 | 100127509 | Diacylglycerol kinase | 3.56 | 2 | 0.798892 | 0.026367716 | Down |  |
| 84 | B6TQW0 | 100279978 | Carboxypeptidase | 22.15 | 9 | 0.794737 | 2.89806E-05 | Down |  |
| 85 | B4FIK0 | 100194404 | Glucose-6-phosphate 1-epimerase | 22.33 | 5 | 0.794271 | 0.022119232 | Down | Glycolysis / Gluconeogenesis |
| 86 | A0A1D6ERH9 | Zm00001d005902 | Sec23/Sec24 protein transport family protein | 11.97 | 7 | 0.792234 | 2.6712E-05 | Down |  |
| 87 | A0A1D6FF44 | 100383086 | Ubiquitinyl hydrolase 1 | 35.78 | 22 | 0.790999 | 6.98406E-06 | Down |  |
| 88 | A0A1D6MSE3 | 103650140 | Dihydrolipoyl dehydrogenase | 64.41 | 27 | 0.790322 | 0.029549726 | Down | Propanoate metabolism // Glyoxylate and dicarboxylate metabolism // Valine, leucine and isoleucine degradation // Pyruvate metabolism // Gluconeogenesis // Citrate cycle // Glycine, serine and threonine metabolism |
| 89 | A0A1D6DX29 | Zm00001d002145 | Formation of crista junctions protein 1 | 22.53 | 9 | 0.782896 | 0.003198567 | Down |  |
| 90 | A0A1D6KV27 | 100285393 | Acylamino-acid-releasing enzyme | 27.26 | 15 | 0.781847 | 0.000187927 | Down |  |
| 91 | A0A1D6J2V0 | Zm00001d024936 | Fatty acid amide hydrolase | 7.37 | 4 | 0.781067 | 0.032964902 | Down |  |
| 92 | K7UCA4 | Zm00001d049764 | Myosin heavy chain-related | 39.25 | 17 | 0.780384 | 0.005082816 | Down |  |
| 93 | C0P429 | 100382295 | UTP--glucose-1-phosphate uridylyltransferase 1 | 28.69 | 12 | 0.777724 | 0.046086958 | Down |  |
| 94 | A0A1D6N1C9 | Zm00001d042088 | Uridine 5'-monophosphate synthase | 23.09 | 14 | 0.765845 | 0.008518478 | Down |  |
| 95 | B6SID3 |  | Uncharacterized protein | 32.76 | 2 | 0.762903 | 0.005675114 | Down |  |
| 96 | C0P7R2 | Zm00001d031071 | Albino or pale green mutant1 | 36.29 | 10 | 0.754294 | 0.000405356 | Down |  |
| 97 | A0A1D6L4K3 | 103644011 | Inosine-5'-monophosphate dehydrogenase | 25.95 | 10 | 0.752488 | 0.002841697 | Down | Purine metabolism |
| 98 | A0A1D6NI54 | Zm00001d044156 | Cytochrome P450 family 72 subfamily A polypeptide 8 | 5.79 | 3 | 0.752096 | 0.048538467 | Down |  |
| 99 | B4F8L9 | 100191382 | Vacuolar protein sorting-associated protein 32 homolog 1 | 26.58 | 5 | 0.750749 | 0.002330993 | Down | Endocytosis |
| 100 | B6TFV6 |  | Charged multivesicular body protein 6 | 23.55 | 6 | 0.745821 | 0.003834951 | Down |  |
| 101 | B6TII5 |  | Pyruvate kinase | 45.42 | 18 | 0.743706 | 0.014311625 | Down | Purine metabolism // Gluconeogenesis // Pyruvate metabolism |
| 102 | C0P6U7 | 100382356 | Uncharacterized protein | 10.66 | 3 | 0.731303 | 0.007401287 | Down |  |
| 103 | B6U543 |  | Versicolorin reductase | 12.6 | 3 | 0.730756 | 0.004941315 | Down | Biosynthesis of unsaturated fatty acids // Biotin metabolism // Monobactam biosynthesis // Fatty acid biosynthesis |
| 104 | B4FAQ2 | 100191932 | Pyridoxal phosphate homeostasis protein | 22.22 | 4 | 0.730408 | 0.037341111 | Down |  |
| 105 | A0A1D6QCK6 | Zm00001d052066 | Mitogen-activated protein kinase kinase 5 | 10.99 | 4 | 0.725107 | 0.026224736 | Down |  |
| 106 | A0A1D6GD81 | 100285036 | NAD(P)-binding Rossmann-fold superfamily protein | 14.13 | 3 | 0.723113 | 0.03048861 | Down |  |
| 107 | B4FFY2 | 100193688 | Zinc finger CCCH domain-containing protein 54 | 18.55 | 4 | 0.722039 | 0.027864438 | Down |  |
| 108 | B6U787 |  | Acetylglutamate kinase | 21.11 | 6 | 0.720171 | 0.026417894 | Down |  |
| 109 | B6TJK4 |  | Glyoxal oxidase | 3.45 | 2 | 0.718341 | 0.041476127 | Down |  |
| 110 | B6SYB8 |  | Pi starvation-induced protein | 46.23 | 3 | 0.718185 | 0.00010454 | Down |  |
| 111 | A0A1D6QH76 | Zm00001d052445 | ARM repeat superfamily protein | 5.24 | 7 | 0.710747 | 0.004973775 | Down |  |
| 112 | B6SJR3 | 100283851 | Mitochondrial import receptor subunit TOM7-1 | 30.38 | 2 | 0.692286 | 0.003770331 | Down |  |
| 113 | B4FSC2 |  | Uncharacterized protein | 24.26 | 4 | 0.666445 | 0.010274763 | Down |  |
| 114 | B6TNT9 | 100283983 | Ubiquilin-1 | 19.7 | 7 | 0.640256 | 0.029566356 | Down | Protein processing in endoplasmic reticulum |
| 115 | C0PH34 | 103627361 | 60S ribosomal protein L6 | 63.01 | 16 | 0.601358 | 0.001348863 | Down | Ribosome |
| 116 | B4FBE5 | 100279891 | Inositol 134-trisphosphate 5/6-kinase 4 | 23.31 | 9 | 0.53829 | 0.012918958 | Down | Inositol phosphate metabolism |
| 117 | A0A1D6LSK8 | Zm00001d036933 | Aldehyde dehydrogenase family 2 member C4 | 5.07 | 2 | 0.364881 | 0.031218568 | Down |  |

^1^ Accession, unique protein identifying number in the UniProt database; ^2^ Gene name/ID; name or ID number of the corresponding gene of the identified DAP as searched against the maize sequence database Gramene ([http://ensemble.gramene.org/Zea mays](http://ensemble.gramene.org/Zea%20mays)); ^3^ Description, annotated biological functions based on Gene Ontology (GO) analysis; ^4^ Covrg. (%), sequence coverage is calculated as the number of amino acids in the peptide fragments observed divided by the protein amino acid length; ^5^ Pept. – peptide fragments, refer to the number of matched peptide fragments generated by trypsin digestion; ^6^ Log2FC - fold change (log 2), is expressed as the ratio of intensities of up-regulated or down-regulated proteins between drought stress treatments and control (well-watered conditions); All the fold change values below 1 represents that the proteins were down-regulated. ^7^ *p* value, statistical significant level (using Student’s *t*-test) < 0.05, ^8^ Expr., gene expression level. Up-, up-regulated; Down- , down-regulated. ^9^ Pathways, metabolic pathways in which the identified protein was found to be significantly enriched.
