# Supplementary material for: Comparative Proteomics and Physiological Analyses Reveal Important Maize Filling-Kernel Drought-Responsive Genes and Metabolic Pathways
Source: Int J Mol Sci. 2019 Jul 31;20(15):3743. doi: 10.3390/ijms20153743 (PMC6696053; doi:10.3390/ijms20153743)
Supplement: Supplementary file 1 [file ijms-20-03743-s001.zip › Supplementary Tables/Supplementary Table 3.docx]

**Supplementary Table 3**. Drought-responsive maize kernel proteins identified overlapping in TC_TD and SC_SD

| No | Accession^1^ | Gene Name/ID^2^ | Description^3^ | Covrg^.4^ | Pept.^5^ | **TC_TD** | | **SC_SD** | | Pathway ^8^ |
| --- | --- | --- | --- | --- | --- | --- | --- | --- | --- | --- |
|  |  |  |  |  |  | Log_2_FC^6^ | p value^7^ | Log_2_FC^6^ | p value^7^ |  |
| 1 | Q9LKL4 | LOX | Lipoxygenase | 51.32 | 41 | 0.829536 | 0.0019284 | 0.347317 | 1.605E-05 | Linoleic acid metabolism |
| 2 | Q946W1 |  | 50kD gamma zein | 37.63 | 8 | 1.362611 | 0.0001324 | 0.714318 | 0.0176877 |  |
| 3 | Q946V2 | 541640 | Legumin 1 | 64.39 | 24 | 1.225667 | 0.0002774 | 1.642167 | 0.0008914 |  |
| 4 | Q84JX6 | Pgk-2 | Phosphoglycerate kinase (Fragment) | 91.97 | 27 | 0.659923 | 0.002592 | 1.552261 | 0.000883 |  |
| 5 | Q6VWJ0 | ccoaomt1 | Caffeoyl-CoA 3-O-methyltransferase 1 | 37.21 | 7 | 0.820266 | 0.0078122 | 1.250517 | 0.0034814 | Phenylalanine metabolism // Flavonoid biosynthesis // Stilbenoid, diarylheptanoid and gingerol biosynthesis // Phenylpropanoid biosynthesis |
| 6 | Q6JB10 | MEG6 | Protein MATERNALLY EXPRESSED GENE 6 | 26.23 | 2 | 0.616716 | 0.003715 | 2.877557 | 0.0009542 |  |
| 7 | Q4W1F6 | trxh2 | Thioredoxin | 70.49 | 10 | 0.575798 | 0.0059801 | 0.793415 | 0.0159092 |  |
| 8 | Q41739 | THI1-2 | Thiamine thiazole synthase 2, chloroplastic | 35.59 | 9 | 1.297086 | 0.0301195 | 1.914151 | 0.0003011 |  |
| 9 | P83506 |  | Probable non-specific lipid-transfer protein 2 | 44.29 | 3 | 1.920009 | 0.0006597 | 1.601063 | 0.0059838 |  |
| 10 | P21641 | OLE18 | Oleosin Zm-II | 42.25 | 5 | 1.688035 | 0.0060549 | 1.598557 | 6.863E-05 |  |
| 11 | O64960 | hsp22 | 23.6 kDa heat shock protein mitochondrial | 42.66 | 9 | 1.325932 | 0.0049955 | 2.948425 | 4.02E-05 | Protein processing in endoplasmic reticulum |
| 12 | K7VW90 | 103635762 | Putative mediator of RNA polymerase II transcription subunit 37c | 60.86 | 37 | 1.43677 | 6.388E-06 | 1.756851 | 0.001174 | Spliceosome // Protein processing in endoplasmic reticulum // Endocytosis |
| 13 | K7VJF3 | 100272911 | Heat shock 70 kDa protein 5 | 58.13 | 38 | 2.155638 | 8.282E-07 | 1.798427 | 7.579E-06 |  |
| 14 | K7VD78 | 100193235 | Putative chaperone clbp family protein | 47.74 | 40 | 1.318359 | 0.0067022 | 1.203269 | 0.0402655 |  |
| 15 | K7UGM3 | Zm00001d054044 | Catalase | 55.67 | 21 | 0.774399 | 0.0009896 | 0.310392 | 2.181E-05 |  |
| 16 | K7U9P3 | Zm00001d049034 | Uncharacterized protein | 42.72 | 3 | 0.713363 | 0.0033536 | 2.414099 | 0.0007992 |  |
| 17 | K7TVZ1 | 100856973 | 6-phosphogluconate dehydrogenase, decarboxylating | 49.9 | 18 | 0.739564 | 0.0197614 | 1.491233 | 0.0369627 | Pentose phosphate pathway // Glutathione metabolism |
| 18 | G4XT43 | P4H4 | Prolyl 4-hydroxylase 4 | 18.25 | 4 | 0.809445 | 0.0014796 | 1.852871 | 0.0001276 | Arginine and proline metabolism |
| 19 | F1DJR5 | PLTZ2 | PLATZ transcription factor (Fragment) | 33.18 | 6 | 0.718194 | 0.0022501 | 4.315461 | 0.0001823 |  |
| 20 | E9JVD4 | AR3 | Aldose reductase | 12.85 | 4 | 1.338454 | 0.0004739 | 1.440567 | 0.0190864 | Glycerolipid metabolism // Pentose and glucuronate interconversions // Gluconeogenesis |
| 21 | C4J410 | 100501536 | Heat shock 70 kDa protein | 62.5 | 40 | 1.372956 | 0.001897 | 1.330634 | 0.0021563 | Protein processing in endoplasmic reticulum // Spliceosome // Endocytosis |
| 22 | C4J093 | Zm00001d044103 | Adenylate kinase 4 | 74.27 | 19 | 0.734681 | 0.0042749 | 0.78217 | 0.0189363 |  |
| 23 | C0PGM3 | Zm00001d033447 | Globulin-1 S allele | 22.03 | 11 | 1.528041 | 0.0013124 | 1.486216 | 0.0020085 |  |
| 24 | C0P8I4 | Zm00001d023243 | 2-isopropylmalate synthase 1 chloroplastic | 81.63 | 41 | 1.274175 | 0.0016139 | 1.548694 | 0.000351 |  |
| 25 | C0P732 | Zm00001d002823 | Hsp70-Hsp90 organizing protein 3 | 45.52 | 29 | 1.303416 | 1.766E-05 | 1.43575 | 4.235E-05 |  |
| 26 | C0P4Q3 | 100196928 | Heat shock protein 90 kDa | 50.7 | 37 | 1.672258 | 0.0011156 | 3.084068 | 6.749E-06 | Plant-pathogen interaction // Protein processing in endoplasmic reticulum |
| 27 | C0HGH4 |  | Uncharacterized protein | 3.46 | 2 | 0.817776 | 0.0025639 | 1.665851 | 0.0116168 |  |
| 28 | C0HFV7 | 100381419 | Apyrase 1 | 17.03 | 7 | 1.335124 | 8.738E-06 | 1.938764 | 1.424E-05 | Purine metabolism // Pyrimidine metabolism |
| 29 | C0HFP5 |  | Uncharacterized protein | 28.16 | 7 | 0.784344 | 0.0039372 | 0.552211 | 0.000138 | Riboflavin metabolism |
| 30 | C0HE67 | 100304261 | Protein WVD2-like 5 | 11.6 | 4 | 0.768296 | 0.0124711 | 1.214526 | 0.0478111 |  |
| 31 | B7ZEQ0 | hsp18 | Small heat-shock protein | 41.67 | 9 | 2.888546 | 6.66E-05 | 3.887358 | 2.504E-05 |  |
| 32 | B6UHW8 |  | Uncharacterized protein | 31.78 | 2 | 0.723276 | 0.0230114 | 2.252909 | 0.0049757 |  |
| 33 | B6UHH1 |  | Uncharacterized protein | 15.97 | 2 | 1.98747 | 3.386E-06 | 2.164095 | 0.000239 |  |
| 34 | B6U237 | Zm00001d009950 | Heat shock 70 kDa protein 14 | 52 | 38 | 1.254599 | 0.0001584 | 1.309131 | 0.0001909 |  |
| 35 | B6TWE1 |  | USP family protein | 43.28 | 7 | 0.761042 | 0.0413127 | 0.670984 | 0.0141329 |  |
| 36 | B6TU39 | 100272496 | Peroxidase | 34.5 | 7 | 0.810433 | 0.0181264 | 0.530236 | 9.037E-05 |  |
| 37 | B6TSW1 |  | Shikimate dehydrogenase | 47.04 | 18 | 0.700817 | 0.0331325 | 1.231294 | 0.0307794 |  |
| 38 | B6TLR1 |  | Vignain | 45.36 | 13 | 0.803629 | 0.004217 | 0.825367 | 0.0027005 |  |
| 39 | B6THZ2 | Zm00001d043390 | Delay of germination 1 | 14.48 | 3 | 1.207596 | 0.0122239 | 1.858569 | 0.0021131 |  |
| 40 | B6THJ8 |  | UDP-glucose 4-epimerase GEPI48 | 25.63 | 7 | 1.205568 | 0.0303217 | 0.62721 | 0.0028862 |  |
| 41 | B6THJ5 | 100283358 | Phosphosulfolactate synthase-related protein | 37.46 | 9 | 1.267668 | 0.0010723 | 1.252089 | 0.0126482 |  |
| 42 | B6TAW1 |  | GAST1 protein | 24.81 | 3 | 1.202028 | 0.001784 | 3.412525 | 1.703E-05 |  |
| 43 | B6T3U2 | 100282123 | CUE domain containing protein | 57.81 | 5 | 1.209268 | 0.008012 | 1.888244 | 0.0013223 |  |
| 44 | B6T3Q3 | 100282113 | Adenine nucleotide alpha hydrolase-like superfamily protein | 31.79 | 5 | 1.224419 | 0.0016764 | 0.447538 | 6.635E-05 |  |
| 45 | B6T339 |  | 17.5 kDa class II heat shock protein | 41.46 | 7 | 1.486299 | 0.0021415 | 2.359381 | 4.797E-05 |  |
| 46 | B6SZ69 | 100281806 | Heat shock cognate 70 kDa protein 2 | 60.25 | 36 | 0.557064 | 0.0046513 | 2.065252 | 0.0001397 | Endocytosis // Protein processing in endoplasmic reticulum // Spliceosome |
| 47 | B6SYQ7 |  | Beta-glucosidase | 16.67 | 7 | 1.258496 | 0.0130625 | 1.64597 | 0.0064669 |  |
| 48 | B6SXF5 |  | Pathogenesis-related protein 1 | 71.25 | 9 | 0.776733 | 0.0002968 | 1.688573 | 0.0005106 |  |
| 49 | B6SWX3 | 100281608 | Anthocyanidin 3-O-glucosyltransferase | 22.61 | 8 | 0.775676 | 0.0402767 | 0.451828 | 0.0001737 |  |
| 50 | B6SUW2 | 100281378 | Alpha/beta-Hydrolases superfamily protein | 16.11 | 5 | 0.804638 | 0.0074419 | 2.134759 | 0.0015919 |  |
| 51 | B6SNQ1 |  | Universal stress protein | 40 | 5 | 0.684224 | 0.0016248 | 2.870314 | 9.517E-07 |  |
| 52 | B6SIX0 | 100280576 | 16.9 kDa class I heat shock protein 1 | 37.66 | 5 | 1.649171 | 0.0018874 | 1.738313 | 0.0005875 | Protein processing in endoplasmic reticulum |
| 53 | B6SIG5 |  | Triosephosphate isomerase, cytosolic | 75.89 | 14 | 0.325277 | 2.51E-05 | 0.687987 | 0.0176561 |  |
| 54 | B6SI42 | 100280594 | Oleosin | 39.2 | 5 | 1.386969 | 0.0029359 | 2.194378 | 0.0015342 |  |
| 55 | B6SGU6 |  | Uncharacterized protein | 40.66 | 3 | 0.817764 | 0.0298777 | 2.663982 | 0.0006937 |  |
| 56 | B6SGF4 | 100280457 | Nonspecific lipid-transfer protein | 31.96 | 3 | 1.205507 | 0.0165585 | 4.535944 | 0.000129 |  |
| 57 | B5AMJ8 | 100285259 | Alpha-1,4 glucan phosphorylase | 50.48 | 35 | 1.201427 | 0.0004288 | 0.680902 | 2.606E-05 | Starch and sucrose metabolism |
| 58 | B4G1Z2 | Zm00001d052651 | Xyloglucan endotransglucosylase/hydrolase | 41.08 | 11 | 0.816977 | 0.0258663 | 0.767398 | 0.0347035 |  |
| 59 | B4G0B0 | 100281936 | Bifunctional inhibitor/lipid-transfer protein/seed storage 2S albumin superfamily protein | 41.18 | 3 | 0.754452 | 0.0425449 | 0.457652 | 1.637E-05 |  |
| 60 | B4FX06 |  | Uncharacterized protein | 10 | 3 | 0.796498 | 0.0265977 | 0.540044 | 0.000605 |  |
| 61 | B4FVX5 |  | Uncharacterized protein | 38.1 | 3 | 0.800173 | 0.014605 | 3.333393 | 0.0004163 |  |
| 62 | B4FUR4 |  | Uncharacterized protein | 39.55 | 11 | 0.802592 | 0.0016174 | 0.621284 | 0.0042778 |  |
| 63 | B4FTP4 | 100273217 | Alcohol dehydrogenase-like 2 | 57.96 | 15 | 0.817133 | 1.867E-05 | 1.2364 | 6.207E-05 | Gluconeogenesis // Tyrosine metabolism // Fatty acid degradation |
| 64 | B4FT59 | Zm00001d047841 | 17.4 kDa class I heat shock protein | 30.06 | 4 | 2.502632 | 0.000162 | 3.128678 | 0.0004726 | Protein processing in endoplasmic reticulum |
| 65 | B4FT54 | Zm00001d039000 | HSP40/DnaJ peptide-binding protein | 31.22 | 7 | 1.291254 | 0.0013263 | 1.370643 | 4.5E-05 |  |
| 66 | B4FSB4 | 100279844 | Leucine-rich repeat (LRR) family protein | 44.28 | 11 | 0.666096 | 0.0036617 | 0.24494 | 6.851E-05 |  |
| 67 | B4FQW0 |  | Stem-specific protein TSJT1 | 31.49 | 5 | 0.726911 | 0.0092722 | 2.843876 | 0.0004627 |  |
| 68 | B4FQ44 | 100272716 | L-tryptophan--pyruvate aminotransferase 1 | 28.54 | 8 | 1.229221 | 0.0002597 | 2.69439 | 4.185E-05 | Tryptophan metabolism |
| 69 | B4FNZ9 |  | General stress protein 39 | 55.92 | 14 | 1.459772 | 0.0001049 | 1.293519 | 0.0034793 |  |
| 70 | B4FLB0 |  | Uncharacterized protein | 61.34 | 30 | 0.557935 | 0.0162876 | 1.72729 | 0.0034008 |  |
| 71 | B4FJ41 | 542263 | Oleosin | 30.77 | 4 | 1.47265 | 0.0030249 | 1.723635 | 0.0042098 |  |
| 72 | B4FFQ0 | 100193620 | Thioredoxin | 61.11 | 5 | 0.694068 | 0.0004236 | 3.213109 | 0.0009846 |  |
| 73 | B4FD80 |  | Uncharacterized protein | 35.98 | 10 | 0.827309 | 0.0034332 | 1.461915 | 0.0051401 |  |
| 74 | B4FAK2 | 100191894 | Shikimate dehydrogenase1 | 48.62 | 18 | 1.301097 | 0.0016449 | 0.525329 | 0.0006304 | Phenylalanine, tyrosine and tryptophan biosynthesis |
| 75 | B4F9K4 | 542723 | 17.5 kDa class II heat shock protein | 48.48 | 8 | 1.337833 | 0.0001577 | 1.570143 | 5.169E-05 | Protein processing in endoplasmic reticulum |
| 76 | B4F9E8 | 100191598 | 17.4 kDa class III heat shock protein | 32.75 | 4 | 1.238583 | 0.0120075 | 0.646856 | 0.000287 | Protein processing in endoplasmic reticulum |
| 77 | A0A1D6PIB7 | 103639475 | Vicilin-like seed storage protein | 39.52 | 18 | 1.397858 | 6.368E-05 | 1.415248 | 0.0068453 |  |
| 78 | A0A1D6NB67 | 100273044 | p-loop containing nucleoside triphosphate hydrolase superfamily protein | 11.26 | 4 | 0.826128 | 0.0044365 | 1.858497 | 0.0221936 |  |
| 79 | A0A1D6N7I4 | 103651028 | Putative mediator of RNA polymerase II transcription subunit 37c | 61.88 | 39 | 2.268889 | 2.825E-05 | 1.967747 | 0.0001481 | Protein processing in endoplasmic reticulum // Spliceosome // Endocytosis |
| 80 | A0A1D6N521 | 100037802 | Lipoxygenase | 47.65 | 34 | 0.771209 | 0.0001822 | 0.698694 | 9.593E-06 | Linoleic acid metabolism |
| 81 | A0A1D6N1Z8 | 541699 | 6-phosphogluconate dehydrogenase, decarboxylating | 64.38 | 23 | 0.798145 | 0.0099359 | 0.633844 | 1.807E-05 | Glutathione metabolism // Pentose phosphate pathway |
| 82 | A0A1D6N0K3 | Zm00001d042022 | Peroxidase | 34.35 | 11 | 0.832533 | 0.0419004 | 0.289448 | 4.896E-05 |  |
| 83 | A0A1D6MSF9 | 100285863 | Alpha-L-fucosidase 2 | 13.72 | 4 | 1.310822 | 0.0001819 | 1.508767 | 0.0041181 |  |
| 84 | A0A1D6MRR5 | 100286246 | Stress-inducible membrane pore protein | 50.29 | 7 | 1.32835 | 0.0001611 | 1.576113 | 3.905E-05 |  |
| 85 | A0A1D6LZ61 | Zm00001d037576 | Pyrophosphate-energized vacuolar membrane proton pump | 18.17 | 14 | 1.316603 | 0.0102325 | 1.72646 | 0.0019965 |  |
| 86 | A0A1D6LXE6 | Zm00001d037384 | Glycosyltransferase | 41.61 | 16 | 0.790394 | 0.0010735 | 0.633785 | 0.0003951 |  |
| 87 | A0A1D6LA47 | 100285900 | Mitochondrial import inner membrane translocase subunit Tim10 | 23.94 | 2 | 0.822507 | 0.0078576 | 0.423108 | 3.203E-05 |  |
| 88 | A0A1D6KZT2 | 100383411 | Plasma membrane ATPase | 26.94 | 22 | 1.305184 | 0.0390739 | 0.74415 | 0.0082554 |  |
| 89 | A0A1D6KNU5 | 100286188 | Temperature-induced lipocalin-1 | 27.86 | 4 | 1.223002 | 0.0003157 | 1.321021 | 9.547E-05 |  |
| 90 | A0A1D6K5C0 | Zm00001d029454 | Sterol-4-alpha-carboxylate 3-dehydrogenase, decarboxylating | 12.17 | 6 | 0.761276 | 0.042092 | 1.317033 | 0.0007032 |  |
| 91 | A0A1D6K128 | 103634588 | Alpha-L-arabinofuranosidase 1 | 7.5 | 4 | 0.79543 | 0.0199881 | 0.353475 | 0.0003277 |  |
| 92 | A0A1D6JKW8 | 107403162 | Nonspecific lipid-transfer protein | 31.63 | 4 | 1.438687 | 0.0004257 | 1.406676 | 0.0008091 |  |
| 93 | A0A1D6JE74 | 103642050 | Uncharacterized protein | 18.95 | 31 | 0.53569 | 0.0397224 | 1.80001 | 0.0091189 |  |
| 94 | A0A1D6I6T8 | 100272368 | Heat shock protein 90-2 | 61.52 | 47 | 1.252531 | 0.0066372 | 0.803252 | 0.0110275 | Protein processing in endoplasmic reticulum // Plant-pathogen interaction |
| 95 | A0A1D6I5B4 | Zm00001d020636 | Cysteine proteinase 2 | 30.21 | 6 | 0.804913 | 0.0029668 | 0.444455 | 0.0002218 |  |
| 96 | A0A1D6HUY4 | 100191940 | AAA-ATPase ASD mitochondrial | 14.2 | 6 | 0.64355 | 0.0217954 | 1.889826 | 0.0056939 |  |
| 97 | A0A1D6H9V5 | 103627237 | Poly [ADP-ribose] polymerase | 17.15 | 7 | 1.703197 | 0.002049 | 1.209482 | 0.0056483 |  |
| 98 | A0A1D6GBB9 | 100193968 | SGS domain-containing protein | 45.09 | 7 | 1.204363 | 3.339E-05 | 1.928983 | 2.353E-05 |  |
| 99 | A0A1D6FGQ6 | 100191407 | TPR repeat-containing thioredoxin TTL1 | 5.23 | 3 | 1.211626 | 0.0396873 | 1.726211 | 0.0133761 |  |
| 100 | A0A1D6FAI6 | Zm00001d007967 | Uncharacterized protein | 23.75 | 2 | 0.732716 | 0.0079048 | 5.425796 | 0.0002812 |  |
| 101 | A0A1D6ED43 | 103646611 | Xanthoxin dehydrogenase | 10.91 | 2 | 0.784758 | 0.0044702 | 0.570981 | 0.0001144 |  |
| 102 | A0A1D6E5Z0 | 100384215 | Phenylalanine ammonia-lyase | 43.1 | 23 | 0.700248 | 0.0011 | 1.481399 | 5.826E-05 |  |
| 103 | A0A1D6DZW5 | Zm00001d002353 | Orf protein | 8.86 | 2 | 0.699086 | 0.0022125 | 1.580536 | 0.001676 |  |
| 104 | A0A096T2V8 | 103648205 | Protein RETICULATA chloroplastic | 15.73 | 5 | 1.231899 | 0.0088758 | 1.203086 | 0.0049306 |  |
| 105 | A0A096RG04 | Zm00001d039387 | SOUL heme-binding family protein | 47.25 | 8 | 1.487888 | 0.0009758 | 1.655745 | 0.0015584 |  |

^1^ Accession, unique protein identifying number in the UniProt database; ^2^ Gene name/ID; name or ID number of the corresponding gene of the identified DAP as searched against the maize sequence database Gramene ([http://ensemble.gramene.org/Zea mays](http://ensemble.gramene.org/Zea%20mays)); ^3^ Description, annotated biological functions based on Gene Ontology (GO) analysis; ^4^ Covrg. (%), sequence coverage is calculated as the number of amino acids in the peptide fragments observed divided by the protein amino acid length; ^5^ Pept. – peptide fragments, refer to the number of matched peptide fragments generated by trypsin digestion; ^6^ Log2FC - fold change (log 2), is expressed as the ratio of intensities of up-regulated or down-regulated proteins between drought stress treatments and control (well-watered conditions); All the fold change values below 1 represents that the proteins were down-regulated. ^7^ *p* value, statistical significant level (using Student’s *t*-test) < 0.05, ^8^ Pathways, metabolic pathways in which the identified protein was found to be significantly enriched.
