# Supplementary material for: Comparative Proteomics and Physiological Analyses Reveal Important Maize Filling-Kernel Drought-Responsive Genes and Metabolic Pathways
Source: Int J Mol Sci. 2019 Jul 31;20(15):3743. doi: 10.3390/ijms20153743 (PMC6696053; doi:10.3390/ijms20153743)
Supplement: Supplementary file 1 [file ijms-20-03743-s001.zip › Supplementary Tables/Supplementary Table 4.docx]

**Supplementary Table 4**. Drought-responsive maize kernel proteins identified specifically in sensitive line MO17

| No | Accession^1^ | Gene Name/ID^2^ | Description^3^ | Covrg.^4^ | Pept.^5^ | Log_2_FC^6^ | p value^7^ | Expr.^8^ | Pathway ^9^ |
| --- | --- | --- | --- | --- | --- | --- | --- | --- | --- |
| 1 | A0A1D6IUP5 | Zm00001d023657 | Uncharacterized protein | 93.62 | 3 | 2.888642 | 0.016637274 | Up |  |
| 2 | B4FS58 | 100275674 | Uncharacterized protein | 30.51 | 3 | 2.494951 | 0.0316284 | Up |  |
| 3 | C0PFC1 |  | Uncharacterized protein | 8.8 | 4 | 2.444345 | 0.025465973 | Up | Steroid biosynthesis |
| 4 | C0PCQ6 | 100383062 | 40S ribosomal protein S2-1 | 65.69 | 18 | 2.011272 | 0.044159417 | Up | Ribosome |
| 5 | B4FS63 | 100274928 | 26S proteasome non-ATPase regulatory subunit 7 homolog A | 45.48 | 11 | 1.972581 | 0.012782659 | Up | Proteasome |
| 6 | A0A1D6IME8 | Zm00001d022414 | Ubiquitin carboxyl-terminal hydrolase 24 | 5.8 | 3 | 1.736028 | 0.033694683 | Up |  |
| 7 | B6TJ22 |  | Glutathione S-transferase parA | 28.57 | 8 | 1.589521 | 0.000321269 | Up | Glutathione metabolism |
| 8 | A0A1D6H397 | Zm00001d015703 | Apoptosis inhibitory protein 5 (API5) | 21.82 | 9 | 1.578287 | 0.0419115 | Up |  |
| 9 | A0A1D6MXI0 | 103650566 | Auxilin-related protein 1 | 4.68 | 2 | 1.573132 | 0.00747973 | Up |  |
| 10 | B6EBQ2 | GH3 | Indole-3-acetic acid amido synthetase | 38.36 | 16 | 1.560798 | 0.040955283 | Up | Plant hormone signal transduction |
| 11 | A0A1D6GLZ6 | Zm00001d013767 | Guanine nucleotide-binding protein-like NSN1 | 16.36 | 6 | 1.553007 | 0.00566343 | Up |  |
| 12 | C0P2Q6 | 100381912 | Glycine-rich RNA-binding protein RZ1C | 6.06 | 2 | 1.545252 | 0.015821882 | Up | Spliceosome |
| 13 | A0A1D6KW35 | Zm00001d033055 | 4-coumarate--CoA ligase-like 7 | 12.86 | 5 | 1.539608 | 0.048300948 | Up |  |
| 14 | A0A1D6GZV1 | 103626594 | DNA polymerase V family | 2.98 | 3 | 1.537846 | 0.047128595 | Up |  |
| 15 | A0A1D6NRA8 | Zm00001d044757 | Dynamin-2A | 29.03 | 25 | 1.532668 | 0.02379562 | Up |  |
| 16 | B4G0Z7 |  | Uncharacterized protein | 41.92 | 4 | 1.523533 | 0.006773407 | Up |  |
| 17 | B4FKN3 | 100216951 | DNA-binding protein HEXBP | 17.24 | 3 | 1.512541 | 0.027178876 | Up |  |
| 18 | B6TYL8 | 100277694 | Uncharacterized protein | 12.36 | 5 | 1.48769 | 0.022422888 | Up |  |
| 19 | A0A1D6EJE6 | 100193501 | Clathrin heavy chain | 52.67 | 78 | 1.482963 | 0.000476277 | Up |  |
| 20 | A0A1D6ENT5 | 103647355 | Putative leucyl-tRNA synthetase | 48.26 | 41 | 1.429513 | 0.019106448 | Up | Aminoacyl-tRNA biosynthesis |
| 21 | A0A1D6G7J2 | 100272808 | Hexosyltransferase | 4.53 | 3 | 1.396476 | 0.049218067 | Up |  |
| 22 | A0A1D6I1D8 | Zm00001d019988 | Homoserine dehydrogenase | 16.47 | 14 | 1.390912 | 0.025406363 | Up |  |
| 23 | A0A1D6FL56 | 100280182 | Putative DUF1296 domain containing family protein | 6.85 | 4 | 1.385474 | 0.018045084 | Up |  |
| 24 | A0A1D6JX34 | 100272589 | Hydroxyproline-rich glycoprotein family protein | 16.96 | 4 | 1.372091 | 0.000196971 | Up |  |
| 25 | A0A1D6GLI6 | 100276857 | U3 snoRNP-associated protein-like EMB2271 | 6.21 | 3 | 1.371044 | 0.017697333 | Up |  |
| 26 | A0A1D6K5R8 | 100501347 | Binding | 2.82 | 2 | 1.367723 | 0.026058525 | Up |  |
| 27 | A0A1D6M007 | 103630125 | Heat shock 70 kDa protein 14 | 39.15 | 32 | 1.366015 | 0.000165238 | Up |  |
| 28 | K7UCD3 | 100501115 | Transmembrane 9 superfamily member | 22.65 | 11 | 1.365737 | 0.00017215 | Up |  |
| 29 | B6U8G0 |  | Uncharacterized protein | 25.6 | 3 | 1.363622 | 0.014427668 | Up |  |
| 30 | Q8LK09 | hda110 | Histone deacetylase | 3.72 | 2 | 1.362944 | 5.14306E-05 | Up |  |
| 31 | A0A1D6FRY9 | Zm00001d010564 | Ypt/Rab-GAP domain of gyp1p superfamily protein | 9.97 | 7 | 1.362211 | 0.020238797 | Up |  |
| 32 | A0A1D6LKJ1 | 100193506 | Thioredoxin superfamily protein | 35.51 | 6 | 1.361761 | 0.005264179 | Up |  |
| 33 | B6UD82 |  | Bifunctional dihydrofolate reductase-thymidylate synthase | 25.05 | 13 | 1.356519 | 0.015917624 | Up | Folate biosynthesis // Pyrimidine metabolism // One carbon pool by folate |
| 34 | C3UZ63 | HSP90-2 | HSP protein | 58.51 | 42 | 1.350648 | 0.00083123 | Up | Plant-pathogen interaction // Protein processing in endoplasmic reticulum |
| 35 | A0A1D6JRW7 | Zm00001d028088 | Ubiquitin system component Cue protein | 1.6 | 2 | 1.345338 | 0.024031923 | Up |  |
| 36 | B4F7V4 |  | Ras-related protein Rab11A | 46.05 | 8 | 1.328293 | 0.027760167 | Up | Endocytosis |
| 37 | A0A1X7YE53 |  | Uncharacterized protein | 53.95 | 17 | 1.31857 | 0.000342261 | Up |  |
| 38 | A0A1D6HXG7 | 100383160 | DEAD-box ATP-dependent RNA helicase 5 | 11.96 | 5 | 1.307015 | 0.030729371 | Up |  |
| 39 | B4FRZ2 | Zm00001d018633 | Pyridoxal 5'-phosphate synthase-like subunit PDX1.2 | 31.23 | 10 | 1.306438 | 0.003468532 | Up |  |
| 40 | B4FIC0 | 100194338 | Citrate synthase | 40.68 | 15 | 1.306133 | 0.001148613 | Up | Glyoxylate and dicarboxylate metabolism |
| 41 | B4FZA1 |  | Mitochondrial glycoprotein | 25.38 | 5 | 1.299222 | 0.000525282 | Up |  |
| 42 | A0A1D6FV20 | 100192799 | Tetratricopeptide repeat (TPR)-like superfamily protein | 17.42 | 6 | 1.299131 | 0.035067207 | Up |  |
| 43 | A0A1D6JW78 | 100857099 | Guanine-nucleotide-exchange protein | 17.36 | 29 | 1.297914 | 0.00033834 | Up |  |
| 44 | B7ZZX5 | 100279781 | Vps51/Vps67 family (Components of vesicular transport) protein | 6.42 | 6 | 1.293606 | 0.000395061 | Up |  |
| 45 | B8A0P7 | 100279928 | Dolichyl-diphosphooligosaccharide--protein glycosyltransferase subunit 2 | 42.26 | 24 | 1.293227 | 0.000551438 | Up | N-Glycan biosynthesis // Protein processing in endoplasmic reticulum |
| 46 | K7VHW8 | Zm00001d047373 | Ubiquitin domain-containing protein DSK2b | 40.25 | 11 | 1.291754 | 0.006859855 | Up |  |
| 47 | B4FIR5 | 100276484 | Peptidase C15 pyroglutamyl peptidase I-like | 24.86 | 3 | 1.291336 | 0.036022282 | Up |  |
| 48 | A0A1D6IL72 | Zm00001d022338 | Phosphatidylinositol 3-and 4-kinase family protein with FAT domain | 4.02 | 13 | 1.289613 | 0.028387009 | Up |  |
| 49 | B6T6P7 |  | UBA and UBX domain-containing protein | 42.81 | 10 | 1.280346 | 0.008731855 | Up | Protein processing in endoplasmic reticulum |
| 50 | A0A1D6MKY7 | Zm00001d039747 | Transcription initiation factor TFIID subunit 15b | 16.45 | 5 | 1.277176 | 0.015086944 | Up |  |
| 51 | A0A1D6N659 | 100384302 | Serine/threonine-protein kinase SRK2A | 20.78 | 7 | 1.276959 | 0.031771689 | Up | MAPK signaling pathway // Plant hormone signal transduction |
| 52 | C0PPD7 | 100384449 | Protein arginine N-methyltransferase PRMT10 | 28.12 | 9 | 1.275458 | 0.002438458 | Up |  |
| 53 | A0A1D6HTJ9 | 100274576 | Peptidyl-prolyl cis-trans isomerase | 16.82 | 3 | 1.274847 | 0.012879938 | Up |  |
| 54 | A0A1D6LAM4 | Zm00001d034758 | Acyl-CoA binding protein | 22.76 | 14 | 1.27427 | 0.037114167 | Up |  |
| 55 | A0A1D6EDK6 | 542319 | Acetyl-CoA carboxylase2 | 39.21 | 77 | 1.271177 | 2.56238E-05 | Up |  |
| 56 | B6TDW6 |  | Cell wall integrity protein scw1 | 19.94 | 6 | 1.270895 | 0.013890483 | Up |  |
| 57 | B4FFA7 | 100193519 | Enoyl-[acyl-carrier-protein] reductase [NADH] chloroplastic | 34.69 | 10 | 1.27012 | 0.001582329 | Up | Biotin metabolism // Monobactam biosynthesis // Fatty acid biosynthesis |
| 58 | B6TMU1 |  | Nascent polypeptide-associated complex subunit beta | 57.99 | 7 | 1.268809 | 0.007444729 | Up |  |
| 59 | A0A1D6DWJ6 | 103645857 | Ubiquitin-associated (UBA)/TS-N domain-containing protein | 4.23 | 2 | 1.267676 | 0.004654466 | Up |  |
| 60 | C4J2B2 | Zm00001d030892 | Uncharacterized protein | 14.29 | 4 | 1.262961 | 0.002461515 | Up |  |
| 61 | C4J6J4 |  | Uncharacterized protein | 39.88 | 13 | 1.261591 | 0.000286818 | Up |  |
| 62 | A0A1D6EYJ6 | 100501049 | HOPM interactor 7 | 15.65 | 24 | 1.260615 | 0.040823434 | Up |  |
| 63 | A0A1D6J9Q7 | Zm00001d025801 | Polyadenylate-binding protein | 40.55 | 20 | 1.25976 | 1.37052E-05 | Up |  |
| 64 | A0A1D6I6D3 | 100272508 | S-adenosyl-L-methionine-dependent methyltransferase superfamily protein | 20.11 | 13 | 1.258841 | 0.005064836 | Up |  |
| 65 | A0A1D6M5I2 | 100274512 | Serine/threonine-protein kinase SRK2A | 33.54 | 9 | 1.252662 | 0.003473667 | Up |  |
| 66 | A0A1D6L9Y9 | Zm00001d034665 | NAD-dependent epimerase/dehydratase | 63.42 | 12 | 1.250582 | 0.00030138 | Up |  |
| 67 | A0A1D6H654 | 103627005 | Uncharacterized protein | 2.28 | 2 | 1.247943 | 0.011995008 | Up |  |
| 68 | A0A1D6LVW5 | 100283814 | Defective in cullin neddylation protein | 12.04 | 3 | 1.244899 | 0.000495404 | Up |  |
| 69 | A0A1D6MU25 | 100501212 | WEB family protein | 8.32 | 4 | 1.243331 | 0.00411855 | Up |  |
| 70 | C0HE46 | 100304252 | THO complex subunit 1 | 8.46 | 5 | 1.240084 | 0.038446163 | Up | RNA transport // Spliceosome |
| 71 | A0A1D6FJI2 | Zm00001d009452 | Putative clathrin assembly protein | 14.21 | 7 | 1.236216 | 0.046264786 | Up |  |
| 72 | A0A1D6HNG1 | Zm00001d018389 | Transmembrane 9 superfamily member | 21.79 | 12 | 1.235761 | 0.000990593 | Up |  |
| 73 | B6TQ43 |  | Reticulon-like protein | 29.68 | 9 | 1.231469 | 0.00215478 | Up |  |
| 74 | B6TAE3 | 101027138 | TPR domain containing protein | 34.13 | 12 | 1.23029 | 0.001356902 | Up |  |
| 75 | A0A1D6HSP6 | 103631960 | Outer envelope protein 61 | 13.19 | 5 | 1.230088 | 0.003765458 | Up |  |
| 76 | B6T6V5 | 100282385 | Ubiquitin carboxyl-terminal hydrolase | 35.77 | 15 | 1.229492 | 0.004869967 | Up |  |
| 77 | B6TRD2 |  | Eukaryotic translation initiation factor 4B | 10.7 | 5 | 1.227445 | 0.025808399 | Up |  |
| 78 | A0A1D6MXN2 | Zm00001d041693 | Golgin candidate 6 | 25.13 | 3 | 1.227397 | 0.001780316 | Up |  |
| 79 | B6UHB1 |  | Uncharacterized protein | 9.95 | 2 | 1.225359 | 0.017956771 | Up |  |
| 80 | A0A1D6MEB3 | 100216801 | Uncharacterized protein | 38.83 | 8 | 1.22526 | 0.006895545 | Up |  |
| 81 | A0A1D6KGE4 | Zm00001d031090 | GTP binding protein | 11.58 | 6 | 1.221595 | 0.003264802 | Up |  |
| 82 | A0A1D6HID6 | 100381905 | Clustered mitochondria protein homolog | 11.5 | 13 | 1.221159 | 0.007732734 | Up |  |
| 83 | C0PEI1 | 100383257 | Exocyst complex component | 7.74 | 5 | 1.219947 | 0.031855543 | Up |  |
| 84 | B4FFK3 |  | Uncharacterized protein | 81.22 | 13 | 1.219876 | 0.006567768 | Up | Endocytosis |
| 85 | B4FZK6 |  | Methionine aminopeptidase | 49.62 | 16 | 1.218957 | 0.002700523 | Up |  |
| 86 | A0A1D6IBD0 | 103633160 | ATP binding protein | 7.83 | 3 | 1.21864 | 0.037035651 | Up |  |
| 87 | A0A1D6H2L6 | 100217072 | Chaperone protein ClpB4 mitochondrial | 29.05 | 23 | 1.218341 | 0.001527557 | Up |  |
| 88 | C0P5T2 |  | Uncharacterized protein | 12.31 | 6 | 1.217269 | 0.022119506 | Up |  |
| 89 | A0A1D6JDF7 | 100192766 | Secretory carrier-associated membrane protein | 41.53 | 5 | 1.216783 | 0.000245193 | Up |  |
| 90 | K7UFV4 | 103654920 | 26S proteasome non-ATPase regulatory subunit 2 homolog A | 42.83 | 34 | 1.215203 | 0.010306175 | Up | Proteasome |
| 91 | A0A1D6IJI0 | Zm00001d022132 | Inter-alpha-trypsin inhibitor heavy chain-related | 10.15 | 2 | 1.214225 | 0.045426874 | Up |  |
| 92 | O65160 | mdJ1 | Chaperone protein dnaJ 3 | 41.05 | 15 | 1.213742 | 0.005875959 | Up | Protein processing in endoplasmic reticulum |
| 93 | A0A1D6LQJ4 | Zm00001d036709 | Transducin/WD40 repeat-like superfamily protein | 12.58 | 3 | 1.211937 | 0.0108212 | Up |  |
| 94 | B6SZV0 |  | Nucleic acid binding protein | 34.01 | 7 | 1.211696 | 0.001643834 | Up |  |
| 95 | A0A1D6HHN9 | 103627622 | Phosphoribulokinase / Uridine kinase family | 9.34 | 5 | 1.211294 | 0.005018257 | Up | Pyrimidine metabolism |
| 96 | A0A1D6N1L5 | 100285224 | RNA-binding (RRM/RBD/RNP motifs) family protein | 23.38 | 5 | 1.210566 | 0.009529787 | Up |  |
| 97 | B4FBP0 | 100272855 | T-complex protein 1 subunit eta | 58.93 | 28 | 1.210234 | 0.000187406 | Up |  |
| 98 | A0A1D6EHK5 | 100384159 | Coatomer subunit beta | 23.32 | 18 | 1.208611 | 0.014119203 | Up |  |
| 99 | A0A1D6HS00 | 103631932 | GYF domain-containing protein | 6.21 | 7 | 1.208383 | 0.017767271 | Up |  |
| 100 | B4FQT3 |  | Uncharacterized protein | 65.17 | 36 | 1.207407 | 0.000831977 | Up |  |
| 101 | B4FJT1 |  | Uncharacterized protein | 6.99 | 3 | 1.207097 | 0.021807156 | Up |  |
| 102 | B4FAD9 | 100191846 | UDP-glucose pyrophosphorylase 2 | 79.92 | 31 | 1.205892 | 0.000706237 | Up | Pentose and glucuronate interconversions // Starch and sucrose metabolism // Amino sugar and nucleotide sugar metabolism |
| 103 | C0P5F2 |  | Uncharacterized protein | 30.43 | 17 | 1.205697 | 0.001120496 | Up |  |
| 104 | B4FUK9 | 100283276 | COP9 signalosome complex subunit 5b | 56.91 | 13 | 1.204554 | 0.032430194 | Up |  |
| 105 | B6TD25 |  | Phosphomevalonate kinase | 11.42 | 3 | 1.203712 | 0.002436478 | Up | Terpenoid backbone biosynthesis |
| 106 | A0A1D6GZG2 | 103626566 | Pectin lyase-like superfamily protein | 19.19 | 7 | 1.203689 | 0.002185945 | Up |  |
| 107 | P29185 | CPN60I | Chaperonin CPN60-1, mitochondrial | 65.68 | 34 | 1.202004 | 0.00039567 | Up | RNA degradation |
| 108 | B6T7S1 |  | Chaperone protein dnaJ 13 | 8.7 | 4 | 1.201672 | 0.016513964 | Up |  |
| 109 | B6SIY7 | 100278212 | Uncharacterized protein | 35.68 | 7 | 1.201594 | 0.000239578 | Up |  |
| 110 | C0PGI4 | Zm00001d018144 | ARM repeat superfamily protein | 32.83 | 15 | 1.201278 | 0.000589314 | Up |  |
| 111 | B6T1U4 |  | AP complex subunit sigma | 32.92 | 5 | 1.200406 | 0.000623891 | Up |  |
| 112 | A0A096PQY2 | 103632349 | Long chain acyl-CoA synthetase 5 | 22.75 | 16 | 0.833048 | 0.00013114 | Down | Peroxisome // Fatty acid biosynthesis and dedradation |
| 113 | B6SL10 | 100280729 | Fasciclin-like arabinogalactan protein 2 | 34.68 | 11 | 0.832799 | 0.002502919 | Down |  |
| 114 | B6SX73 | 100282133 | 60S ribosomal protein L35 | 46.34 | 9 | 0.832139 | 0.004341226 | Down | Ribosome |
| 115 | B6TVY0 | 100278608 | Mitochondrial ribosomal protein L27 | 28 | 3 | 0.828079 | 0.024456576 | Down |  |
| 116 | A0A1D6EBS5 | 542342 | Starch branching enzyme IIa | 45.82 | 29 | 0.826349 | 0.001147648 | Down |  |
| 117 | A0A1D6J5X9 | Zm00001d025258 | Dihydrolipoyllysine-residue succinyltransferase component of 2-oxoglutarate dehydrogenase complex | 58.9 | 18 | 0.826006 | 0.007786067 | Down |  |
| 118 | A0A1D6QRF4 | Zm00001d053674 | Tubulin binding cofactor C domain-containing protein | 13.59 | 7 | 0.825295 | 0.03075346 | Down |  |
| 119 | B6UH19 |  | 60S ribosomal protein L32 | 56.39 | 8 | 0.824755 | 0.018986291 | Down |  |
| 120 | B4F8E3 | 100191322 | Electron transfer flavoprotein subunit alpha mitochondrial | 33.62 | 8 | 0.82471 | 0.002547119 | Down |  |
| 121 | B8A377 | 542438 | Cysteine synthase | 58.46 | 15 | 0.823527 | 0.003223152 | Down | Cysteine and methionine metabolism // Sulfur metabolism |
| 122 | B6TXX0 | Zm00001d017833 | Ribose-phosphate pyrophosphokinase 4 | 50.77 | 13 | 0.823168 | 0.00150595 | Down |  |
| 123 | A0A1D6Q3F3 | 100272327 | CLP protease regulatory subunit CLPX3 mitochondrial | 9.78 | 5 | 0.822941 | 0.002047599 | Down |  |
| 124 | B4FJJ9 | 100216749 | ATP-dependent (S)-NAD(P)H-hydrate dehydratase | 26.63 | 9 | 0.822374 | 0.00084165 | Down |  |
| 125 | B6TE57 |  | IQ calmodulin-binding motif family protein | 24.76 | 11 | 0.820611 | 0.000833559 | Down |  |
| 126 | A0A1D6IJS9 | 100281249 | Ankyrin repeat family protein | 11.06 | 2 | 0.820443 | 0.00629941 | Down |  |
| 127 | B6SNW4 | 100280887 | Glutamine amidotransferase subunit pdxT | 15.69 | 3 | 0.819952 | 0.008740628 | Down | Vitamin B6 metabolism |
| 128 | C0P2E6 | 100192869 | Hypersensitive-induced reaction protein 4 | 29.14 | 5 | 0.819735 | 0.000287885 | Down |  |
| 129 | B4FBY6 |  | Uncharacterized protein | 45.77 | 16 | 0.819082 | 0.042539277 | Down |  |
| 130 | B6TCK3 | 103649684 | NADH-cytochrome b5 reductase | 59.49 | 15 | 0.818471 | 0.000114767 | Down | Amino sugar and nucleotide sugar metabolism |
| 131 | B4FDI5 | 100193022 | Bifunctional monothiol glutaredoxin-S16 chloroplastic | 16.84 | 5 | 0.816708 | 0.006876948 | Down |  |
| 132 | B4FVC7 |  | Uncharacterized protein | 10.07 | 2 | 0.815628 | 0.045174941 | Down |  |
| 133 | B6TVN0 | 100284613 | 40S ribosomal protein S16 | 44.3 | 8 | 0.814765 | 0.003049468 | Down | Ribosome |
| 134 | Q9SAZ6 | Ppc1C | Phosphoenolpyruvate carboxylase | 59.48 | 54 | 0.813837 | 0.000295519 | Down | Carbon fixation in photosynthetic organisms // Pyruvate metabolism |
| 135 | B6T7B2 | 100282862 | 40S ribosomal protein S9 | 47.94 | 11 | 0.811399 | 0.005123917 | Down | Ribosome |
| 136 | B4FID1 | 100194345 | 40S ribosomal protein S17-1 | 63.38 | 11 | 0.811333 | 0.012965772 | Down | Ribosome |
| 137 | B4FGJ9 | 100193861 | Chaperone DnaJ-domain superfamily protein | 14.61 | 4 | 0.806996 | 0.044760152 | Down |  |
| 138 | Q94G04 |  | Diphosphonucleotide phosphatase 1 | 27.04 | 8 | 0.805621 | 0.003345977 | Down |  |
| 139 | A0A1D6INP6 | Zm00001d022510 | 4-alpha-glucanotransferase DPE2 | 29.92 | 27 | 0.804993 | 0.000196726 | Down |  |
| 140 | B6T2J0 |  | Thioredoxin | 41.41 | 6 | 0.804804 | 0.006053483 | Down |  |
| 141 | B6U0D3 |  | Uncharacterized protein | 41.73 | 10 | 0.804701 | 6.61568E-05 | Down |  |
| 142 | A0A1D6LWP3 | 100280420 | Ketose-bisphosphate aldolase class-II family protein | 38.71 | 45 | 0.803834 | 0.010007221 | Down |  |
| 143 | B6TE40 |  | 60S ribosomal protein L22-2 | 46.46 | 6 | 0.803694 | 3.40341E-05 | Down | Ribosome |
| 144 | B4FJY6 |  | Uncharacterized protein | 14.83 | 3 | 0.802221 | 0.00139697 | Down |  |
| 145 | C0P522 |  | Uncharacterized protein | 5.33 | 2 | 0.802178 | 0.036554601 | Down |  |
| 146 | A0A1D6M1A3 | 103630192 | Uncharacterized protein | 17.65 | 14 | 0.80154 | 8.32753E-05 | Down |  |
| 147 | A0A1D6IP64 | 100275036 | AMP deaminase | 15.01 | 12 | 0.800003 | 0.001055252 | Down | Purine metabolism |
| 148 | B6SHU5 | 100281475 | Fasciclin-like arabinogalactan protein 7 | 23.42 | 6 | 0.795931 | 3.74297E-06 | Down |  |
| 149 | A0A1D6LXJ5 | Zm00001d037401 | Proteasome activating protein 200 | 4.53 | 7 | 0.792355 | 0.033688719 | Down |  |
| 150 | A0A1D6K1V1 | Zm00001d029034 | ABC transporter B family member 25 mitochondrial | 17.77 | 9 | 0.791595 | 8.15665E-06 | Down |  |
| 151 | B6TE60 | 100276561 | Putative proteasome inhibitor | 21.59 | 5 | 0.786656 | 0.003245002 | Down | Proteasome |
| 152 | B4FH14 | Zm00001d019233 | Vacuolar protein sorting-associated protein 2 homolog 1 | 17.94 | 5 | 0.781711 | 0.004726528 | Down |  |
| 153 | C0PI69 |  | Uncharacterized protein | 11.26 | 3 | 0.77888 | 0.000301188 | Down |  |
| 154 | B6T828 | 100282495 | Grx_C4-glutaredoxin subgroup I | 48.89 | 5 | 0.778056 | 0.019166447 | Down |  |
| 155 | A0A1D6L5P9 | 100382459 | Peroxidase | 16.79 | 4 | 0.777592 | 0.000229538 | Down |  |
| 156 | B6TIR4 |  | Ribulose-phosphate 3-epimerase | 19.56 | 3 | 0.770891 | 0.008268384 | Down | Pentose phosphate pathway // Carbon fixation in photosynthetic organisms // Pentose and glucuronate interconversions |
| 157 | B6SSX2 | 100281180 | Gamma-glutamyltranspeptidase 1 | 17.93 | 7 | 0.768872 | 0.00970393 | Down | Taurine and hypotaurine metabolism // Glutathione metabolism // Cyanoamino acid metabolism |
| 158 | B8A0Q3 | 100279929 | Carboxypeptidase | 19.57 | 8 | 0.768758 | 0.002294614 | Down |  |
| 159 | B6UDC2 | 100192950 | Actin-interacting protein 1-2 | 38.52 | 20 | 0.763565 | 0.007642228 | Down |  |
| 160 | B6TNG9 |  | Aminoacylase-1 | 25.97 | 8 | 0.762332 | 0.00140588 | Down |  |
| 161 | B6UCV5 | 100285997 | Shikimate biosynthesis protein aroDE | 49.51 | 19 | 0.762282 | 5.91186E-05 | Down | Phenylalanine, tyrosine and tryptophan biosynthesis |
| 162 | K7VP16 | 100276764 | Protein RETICULATA-RELATED 3 chloroplastic | 15.16 | 5 | 0.761498 | 0.001360439 | Down |  |
| 163 | B6TBM1 | 100282813 | Alpha-soluble NSF attachment protein | 60.21 | 16 | 0.760727 | 0.000385729 | Down |  |
| 164 | A0A1D6JVV6 | 103633550 | Filament-like plant protein 4 | 3.3 | 4 | 0.746174 | 0.001086699 | Down |  |
| 165 | A0A1D6PWU0 | 100502233 | Succinate dehydrogenase2 | 42.56 | 8 | 0.743791 | 0.011626389 | Down |  |
| 166 | A0A1D6FPN2 | Zm00001d010179 | Putative subtilase family protein | 7.46 | 4 | 0.737701 | 0.003647789 | Down |  |
| 167 | B4FY36 | 100284444 | Caltractin | 16.86 | 2 | 0.736457 | 0.003835754 | Down | Plant-pathogen interaction |
| 168 | B6TD69 |  | 40S ribosomal protein S17-4 | 63.38 | 11 | 0.734036 | 0.007271132 | Down |  |
| 169 | B6TPA4 | 100284027 | Carnitine racemase/ catalytic | 39.67 | 8 | 0.730991 | 0.018970543 | Down |  |
| 170 | A0A1D6L9A0 | 100193263 | 40S ribosomal protein S26 | 50.76 | 5 | 0.730959 | 0.026177308 | Down | Ribosome |
| 171 | B4G004 | 100274288 | Beta-glucosidase 44 | 32.87 | 15 | 0.729781 | 0.000165857 | Down | Starch and sucrose metabolism // Cyanoamino acid metabolism // Phenylpropanoid biosynthesis |
| 172 | Q8W0V2 | 542495 | Lipoxygenase | 53.01 | 38 | 0.729404 | 0.000122936 | Down | Linoleic acid metabolism |
| 173 | B6T0A4 |  | 4-hydroxy-4-methyl-2-oxoglutarate aldolase | 23.21 | 4 | 0.723059 | 0.017645558 | Down |  |
| 174 | B4FVT1 | 100273479 | Peroxidase | 6.44 | 3 | 0.718297 | 0.024683491 | Down | Phenylpropanoid biosynthesis |
| 175 | A0A1D6IJ82 | Zm00001d022108 | Uncharacterized protein | 13.16 | 2 | 0.715877 | 0.026411846 | Down |  |
| 176 | A0A1D6MLR2 | 100382383 | Protein ASPARTIC PROTEASE IN GUARD CELL 1 | 11.26 | 5 | 0.715078 | 2.94321E-05 | Down |  |
| 177 | B6T9W6 |  | Charged multivesicular body protein 1b | 7.84 | 2 | 0.71326 | 0.013687464 | Down |  |
| 178 | P80607 | UPTG | Probable UDP-arabinopyranose mutase 1 | 77.75 | 26 | 0.697619 | 0.027624217 | Down | Amino sugar and nucleotide sugar metabolism |
| 179 | C0PCX9 |  | Mg-protoporphyrin IX chelatase | 4.84 | 2 | 0.693929 | 0.023184427 | Down |  |
| 180 | A0A1D6PFB7 | 100273042 | LisH and RanBPM domains containing protein | 15.94 | 2 | 0.686667 | 0.045278861 | Down |  |
| 181 | A0A1D6MYR1 | 100216783 | Switch/sucrose nonfermenting 3C | 3.83 | 2 | 0.675905 | 0.022342429 | Down |  |
| 182 | A0A1D6FJH2 | 103635288 | Double-stranded DNA-binding family protein | 22.52 | 3 | 0.651055 | 0.012489538 | Down |  |
| 183 | C0HHJ5 | 100192575 | 2-oxoglutarate (2OG) and Fe(II)-dependent oxygenase superfamily protein | 9.73 | 3 | 0.650842 | 0.028971531 | Down |  |
| 184 | B6SQD4 | 100280962 | Catalytic/ oxidoreductase, acting on NADH or NADPH | 26.37 | 2 | 0.625053 | 0.040240694 | Down |  |
| 185 | C4J0N7 |  | Uncharacterized protein | 12.6 | 5 | 0.609116 | 0.008061958 | Down |  |
| 186 | B6TRW9 |  | Chlorophyll a-b binding protein, chloroplastic | 23.58 | 2 | 0.352542 | 0.047224504 | Down |  |

^1^ Accession, unique protein identifying number in the UniProt database; ^2^ Gene name/ID; name or ID number of the corresponding gene of the identified DAP as searched against the maize sequence database Gramene ([http://ensemble.gramene.org/Zea mays](http://ensemble.gramene.org/Zea%20mays)); ^3^ Description, annotated biological functions based on Gene Ontology (GO) analysis; ^4^ Covrg. (%), sequence coverage is calculated as the number of amino acids in the peptide fragments observed divided by the protein amino acid length; ^5^ Pept. – peptide fragments, refer to the number of matched peptide fragments generated by trypsin digestion; ^6^ Log2FC - fold change (log 2), is expressed as the ratio of intensities of up-regulated or down-regulated proteins between drought stress treatments and control (well-watered conditions); All the fold change values below 1 represents that the proteins were down-regulated. ^7^ *p* value, statistical significant level (using Student’s *t*-test) < 0.05, ^8^ Expr., gene expression level. Up-, up-regulated; Down- , down-regulated. ^9^ Pathways, metabolic pathways in which the identified protein was found to be significantly enriched.
