# Supplementary material for: Comparative Proteomics and Physiological Analyses Reveal Important Maize Filling-Kernel Drought-Responsive Genes and Metabolic Pathways
Source: Int J Mol Sci. 2019 Jul 31;20(15):3743. doi: 10.3390/ijms20153743 (PMC6696053; doi:10.3390/ijms20153743)
Supplement: Supplementary file 1 [file ijms-20-03743-s001.zip › Supplementary Tables/Supplementary Table 5.docx]

**Supplementary Table 6** Protein – Protein Interaction Analysis of the YE8112 Drought Responsive DAPs

| **Cluster/pair** | **Protein/Gene ID** | **Description** | **Broad biological function** |
| --- | --- | --- | --- |
| **I** | B6SID7 | Late embryogenesis abundant protein, group 3 |  |
|  | B4FT54 | HSP40/DnaJ peptide-binding protein | chaperone binding // chaperone binding // chaperone cofactor-dependent protein refolding |
|  | A0A1D6N7I4 | Putative mediator of RNA polymerase II transcription subunit 37c | ATP binding // heat shock protein binding // misfolded protein binding // protein folding chaperone// cellular response to heat // cellular response to unfolded protein |
|  | K7VW90 | Putative mediator of RNA polymerase II transcription subunit 37c | ATP binding // heat shock protein binding // misfolded protein binding // protein folding chaperone// cellular response to heat // cellular response to unfolded protein |
|  | B6SZ69 | Heat shock cognate 70 kDa protein 2 | ATP binding // heat shock protein binding // misfolded protein binding // protein folding chaperone// cellular response to heat // cellular response to unfolded protein |
|  | A0A1D6I6T8 | Heat shock protein 90-2 | ATP binding // unfolded protein binding // protein folding |
|  | K7VJF3 | Heat shock 70 kDa protein 5 | ATP binding // heat shock protein binding // misfolded protein binding // protein folding chaperone// cellular response to heat // cellular response to unfolded protein |
|  | B6U237 | Heat shock 70 kDa protein 14 | ATP binding |
|  | C0P732 | Hsp70-Hsp90 organizing protein 3 | Hsp90 protein binding |
|  | O64960 | 23.6 kDa heat shock protein mitochondrial | unfolded protein binding // response to heat // response to osmotic stress |
| **II** | A0A1D6LPQ2 | Glycosyl hydrolase family 31 protein | carbohydrate binding // hydrolase activity, hydrolyzing O-glycosyl compounds // carbohydrate metabolic process |
|  | C0HGR4 | 4-alpha-glucanotransferase | 4-alpha-glucanotransferase activity // beta-maltose 4-alpha-glucanotransferase activity |
|  | A0A1D6I6A1 | Isoamylase-type starch debranching enzyme3 | hydrolase activity, hydrolyzing O-glycosyl compounds |
|  | K7VJE7 | Starch branching enzyme III | 1,4-alpha-glucan branching enzyme activity // hydrolase activity, hydrolyzing O-glycosyl compounds |
|  | Q41740 | 1,4-alpha-glucan branching enzyme | 1,4-alpha-glucan branching enzyme activity // hydrolase activity, hydrolyzing O-glycosyl compounds // cation binding |
|  | A0A1D6INP5 | 4-alpha-glucanotransferase DPE2 | 4-alpha-glucanotransferase activity // starch binding |
|  | B5AMJ8 | Alpha-1,4 glucan phosphorylase | glycogen phosphorylase activity |
|  | B6TDF8 | Glyceraldehyde-3-phosphate dehydrogenase | NAD binding // NADP binding // glucose metabolic process |
| **Protein pair I** | B6SGF3 | Glyoxalase family protein superfamily |  |
|  | B4FQG0 | Hydrogen peroxide-induced 1 |  |
| **Protein pair II** | K7TVZ1 | 6-phosphogluconate dehydrogenase, decarboxylating | NADP binding // phosphogluconate 2-dehydrogenase activity // phosphogluconate dehydrogenase (decarboxylating) activity // response to abscisic acid |
|  | A0A1D6N1Z8 | 6-phosphogluconate dehydrogenase, decarboxylating | NADP binding // phosphogluconate 2-dehydrogenase activity // phosphogluconate dehydrogenase (decarboxylating) activity // response to abscisic acid |

**I-** LEA protein group 3, DnaJ peptide-binding protein, HSP 90-2, Putative mediator of RNA polymerase II transcription subunit 37c: Higher HSPs

**II-** Glycosyl hydrolase family 31 protein, 4-alpha-glucanotransferase, Isoamylase-type starch debranching enzyme3, Starch branching enzyme III, 4-alpha-glucanotransferase DPE2, Alpha-1,4 glucan phosphorylase, Glyceraldehyde-3-phosphate dehydrogenase.

**Protein pair I-**  Glyoxalase family protein superfamily, Hydrogen peroxide-induced 1.

**Protein pair II-** 6-phosphogluconate dehydrogenase, decarboxylating.

**NOTES**

1. Heat shock proteins act as molecular chaperones that are responsible for protein synthesis, folding, targeting assembly, translocation and degradation in many normal cellular processes and involved in proteins and membrane stabilization as well as assisting protein refolding under stress
2. Initially, late embryogenesis abundant (LEA) proteins were characterized in seed but they are also present in vegetative tissue. LEA gene is induced either through ABA application or through environmental stress such as, dehydration, osmotic and low temperature stresses. LEA is associated with dehydration tolerance and whole plant resistance to drought, salt and cold stress. It has been suggested that they act as a water holding molecules in ion sequestration and have the ability of membrane and protein stabilization .
3. HSP70 - molecular chaperones regulating the folding and accumulation of proteins as well as localization and degradation.
4. HSP70 - In almost all organisms, the Hsp70 functions as chaperones for newly synthesized proteins to prevent their accumulations as aggregates and folds in a proper way during their transfer to their final location.
5. HSP 90 - the class Hsp90 shares with other classes, the role being molecular chaperones as Hsp90 can bind Hsp70 in many chaperone complexes and has important role in signaling protein function and trafficking.
